# Regulation of *Atp7a* RNA contributes to differentiation-dependent Cu redistribution in skeletal muscle cells

**DOI:** 10.1093/mtomcs/mfad042

**Published:** 2023-06-30

**Authors:** Thomas J Whitlow, Yu Zhang, Nathan Ferguson, Alexandra M Perez, Hemchandra Patel, Josephine A Link-Kemp, Ethan M Larson, Allison T Mezzell, Vinit C Shanbhag, Michael J Petris, Katherine E Vest

**Affiliations:** Department of Molecular and Cellular Biosciences, University of Cincinnati College of Medicine, Cincinnati, OH, USA; Department of Molecular and Cellular Biosciences, University of Cincinnati College of Medicine, Cincinnati, OH, USA; Department of Molecular and Cellular Biosciences, University of Cincinnati College of Medicine, Cincinnati, OH, USA; Department of Molecular and Cellular Biosciences, University of Cincinnati College of Medicine, Cincinnati, OH, USA; Department of Molecular and Cellular Biosciences, University of Cincinnati College of Medicine, Cincinnati, OH, USA; Department of Molecular and Cellular Biosciences, University of Cincinnati College of Medicine, Cincinnati, OH, USA; Department of Molecular and Cellular Biosciences, University of Cincinnati College of Medicine, Cincinnati, OH, USA; Department of Molecular and Cellular Biosciences, University of Cincinnati College of Medicine, Cincinnati, OH, USA; Department of Ophthalmology, Department of Biochemistry, University of Missouri School of Medicine, Columbia, MO, USA; Department of Ophthalmology, Department of Biochemistry, University of Missouri School of Medicine, Columbia, MO, USA; Department of Molecular and Cellular Biosciences, University of Cincinnati College of Medicine, Cincinnati, OH, USA

**Keywords:** copper, ATP7A, myogenesis, RNA stability, lysyl oxidase

## Abstract

Cu (Cu) is essential for several biochemical pathways due to its role as a catalytic cofactor or allosteric regulator of enzymes. Its import and distribution are tightly controlled by transporters and metallochaperones and Cu homeostasis is maintained by balancing Cu uptake and export. Genetic diseases are caused by impaired Cu transporters CTR1, ATP7A, or ATP7B but little is known about the regulatory mechanisms by which these proteins meet the fluctuating demands of Cu in specific tissues. Cu is required for differentiation of skeletal myoblasts to myotubes. Here, we demonstrate that ATP7A is needed for myotube formation and that its increased abundance during differentiation is mediated by stabilization of *Atp7a* mRNA via the 3′ untranslated region. Increased ATP7A levels during differentiation resulted in increased Cu delivery to lysyl oxidase, a secreted cuproenzyme that needed for myotube formation. These studies identify a previously unknown role for Cu in regulating muscle differentiation and have broad implications for understanding Cu-dependent differentiation in other tissues.

## Graphical Abstract

**Graphical Abstract:**
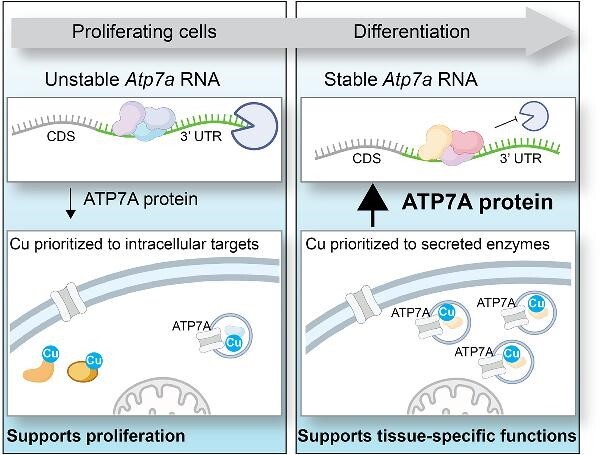
Copper is an essential but toxic trace metal nutrient that must be maintained within a tight homeostatic range. Here, we demonstrate that copper is prioritized to different targets depending on differentiation state in skeletal myoblasts and that post-transcriptional regulation of the trans-Golgi copper exporter ATP7A contributes to copper redistribution.

## Introduction

Mammalian tissues undergo substantial growth and remodeling during regeneration, which echoes similar processes during late fetal and early postnatal growth. Tissue regeneration employs endogenous pools of stem cells and requires fluctuating morphologic and metabolic changes governed by precisely timed gene expression. Given that a multitude of metabolic enzymes and signaling components require trace metal nutrients as catalytic cofactors or allosteric regulators, temporal regulation of trace metal distribution is likely to be essential for normal tissue remodeling. Copper (Cu) is an essential trace nutrient that acts as both enzyme cofactor and allosteric regulator of cell signaling.^1^ Due to its inherent toxicity, Cu levels and distribution must be tightly controlled and aberrant Cu handling leads to disease.^2,3^ The essential machinery governing Cu homeostasis in mammalian cells is known, but how regulation of this machinery contributes to tissue-specific needs is poorly understood. Understanding regulated Cu distribution in different tissues is especially important considering the pleiotropic defects in different tissues observed in individuals with Menkes disease, Wilson disease, and other disorders of Cu dyshomeostasis.

Cu is required for the activity of several enzymes including cytochrome c oxidase, superoxide dismutase, and extracellular multicopper oxidases like ceruloplasmin and hephaestin. More recent studies have revealed that Cu can act as an allosteric modifier of multiple kinases including MEK, ULK1, CK2, and PDK1.^4–7^ These kinases are needed for fundamental processes like cell proliferation, survival, and autophagy and are important for both normal cell growth and tumorigenesis. Cu can also promote the activity of E2D ubiquitin ligases and influence ubiquitylation and proteostasis.^8^ Conversely, Cu binding can inhibit enzyme activity in the case of the phosphodiesterase PDE3B, thus promoting cyclic AMP or cyclic adenosine monophosphate (cAMP)-dependent lipolysis.^9^ Despite these essential functions, Cu is toxic if concentrations exceed normal levels. Excess Cu can inhibit metalloproteins by outcompeting their normal metal cofactor for binding or may disrupt the overall redox balance in the cell.^10,11^ Elevated mitochondrial Cu leads to cuproptosis, a unique form of cell death that is mediated by Cu-induced aggregation of lipoylated tricarboxylic acid cycle (TCA) cycle proteins.^12^ Thus, Cu import, distribution, and storage must be tightly controlled.

Cu homeostasis is maintained by a series of membrane transporters and soluble Cu-binding molecules such as metallochaperones and metabolites. Cu is imported to the cytoplasm by CTR1 where it binds to glutathione,^13^ metallochaperones, or unidentified small molecules.^14,15^ The metallochaperone Cu-chaperone for SOD1 (CCS) delivers Cu to its major target SOD1 as well as other proteins including MEK1/2.^16,17^ ATOX1 is a related metallochaperone that also mediates MEK1/2 metalation and facilitates Cu entry to the secretory pathway through Cu delivery to the Cu exporters ATP7A and ATP7B.^18,19^ Cu is delivered into the mitochondrial matrix by SLC25A3 for subsequent assembly into cytochrome c oxidase in the intermembrane space.^20^ Cysteine-rich metallothionein proteins contribute to intracellular Cu buffering, but the most important mechanism of Cu detoxification is export via ATP7A and ATP7B.^21^ These Cu-transporting ATPases normally reside within the trans-Golgi network to deliver Cu to secreted Cu enzymes but excess Cu stimulates ATP7A/B trafficking to post-Golgi vesicles and the plasma membrane to mediate Cu export.^22,23^ The importance of ATP7A in Cu detoxification is highlighted by the fact that loss of ATP7A more potently sensitizes cells to Cu toxicity than does metallothionein deficiency.^21^ Thus, the Cu-transporting ATPases are critical mediators of intracellular Cu homeostasis.

Despite progress in identifying the key components of Cu homeostasis, the regulatory mechanisms that ensure appropriate Cu is available to meet the fluctuating demands of tissue development and repair remain poorly understood. Due to their tractable nature *in vitro* and *in vivo*, muscle stem cells and stem-cell derived myoblasts are commonly used to model mammalian tissue development and regeneration. Muscle stem cells contribute to both early postnatal growth and regeneration of damaged muscle tissue^24^ and provide a model for studying Cu import and distribution in skeletal muscle^25,26^ which contains approximately 23% of total systemic Cu.^27^ Cu deficiency is rate limiting for the differentiation of myoblasts into myotubes, a process that coincides with increased levels of the high affinity Cu importer CTR1 and the trans-Golgi Cu transporter ATP7A.^26^ Moreover, loss-of-function mutations in ATP7A are known to produce developmental defects in patients with Menkes disease, including delayed muscle development and hypotonia. While these observations suggest a role for ATP7A in muscle development, a mechanistic understanding is currently lacking.

Here, we sought to better understand the regulation of ATP7A in the context of myoblast differentiation. We found that the differentiation of immortalized C2C12 myoblasts into myotubes was dependent on Cu, which induced a steady-state increase in ATP7A protein abundance. This process was associated with a Cu-dependent increase in *Atp7a* RNA stability beginning in the early stages of differentiation. In proliferating myoblasts, but not myotubes, the *Atp7a* 3′ untranslated region (UTR) was found to suppress luciferase expression when tethered to the 3′end of luciferase mRNA and was derepressed by exogenous Cu. Silencing of ATP7A in C2C12 myoblasts or primary mouse myoblasts impaired myotube formation and reduced activity of extracellular lysyl oxidase (LOX), an enzyme previously shown to function in muscle development.^28,29^ This defect in ATP7A-deficient myoblast differentiation was ameliorated by exogenous LOX and lysyl oxidase like-2 (LOXL2). Taken together, these studies identify post-transcriptional regulation of ATP7A-dependent LOX activity as a novel control point for muscle differentiation that may broadly apply to other forms of Cu-dependent tissue development and repair.

## Results

### C2C12 myoblast differentiation is associated with increased levels of Cu and ATP7A expression

**Fig. 1:**
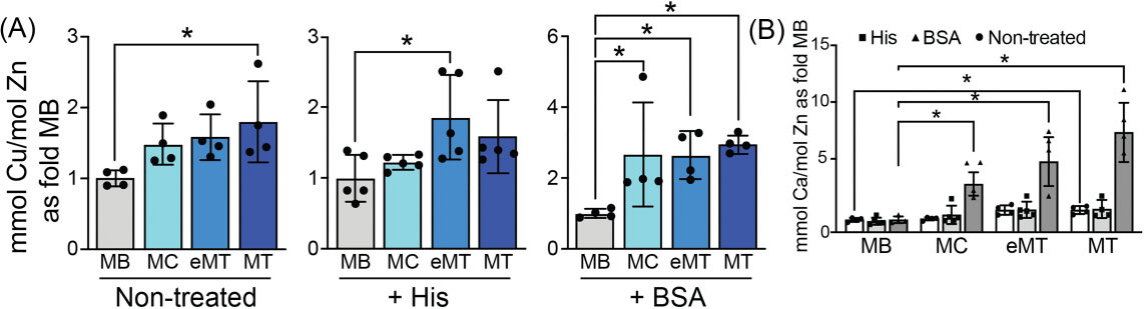
**Cu increases during C2C12 differentiation.** (A) Levels of Cu (Cu) normalized to zinc (Zn) from C2C12 in myoblasts (MB), myocytes (MC), early myotubes (eMTs), and mature myotubes (MT) in normal growth or differentiation medium (Nontreated), in the presence of 20 µM histidine (+ His) or 10% bovine serum albumin (+ BSA) showing a significant increase in Cu for all differentiating samples. Values were measured using ICP–OES and are reported as fold change of the average for each MB sample. (B) Levels of calcium (Ca) as a positive control normalized to Zn for all three treatments reported as fold of MB showing an increase in all samples. Shown is the mean ± standard deviation for *n* = 4–5 experiments. For all experiments, statistical significance was determined using one-way ANOVA (*P < 0.05).

**Fig. 2:**
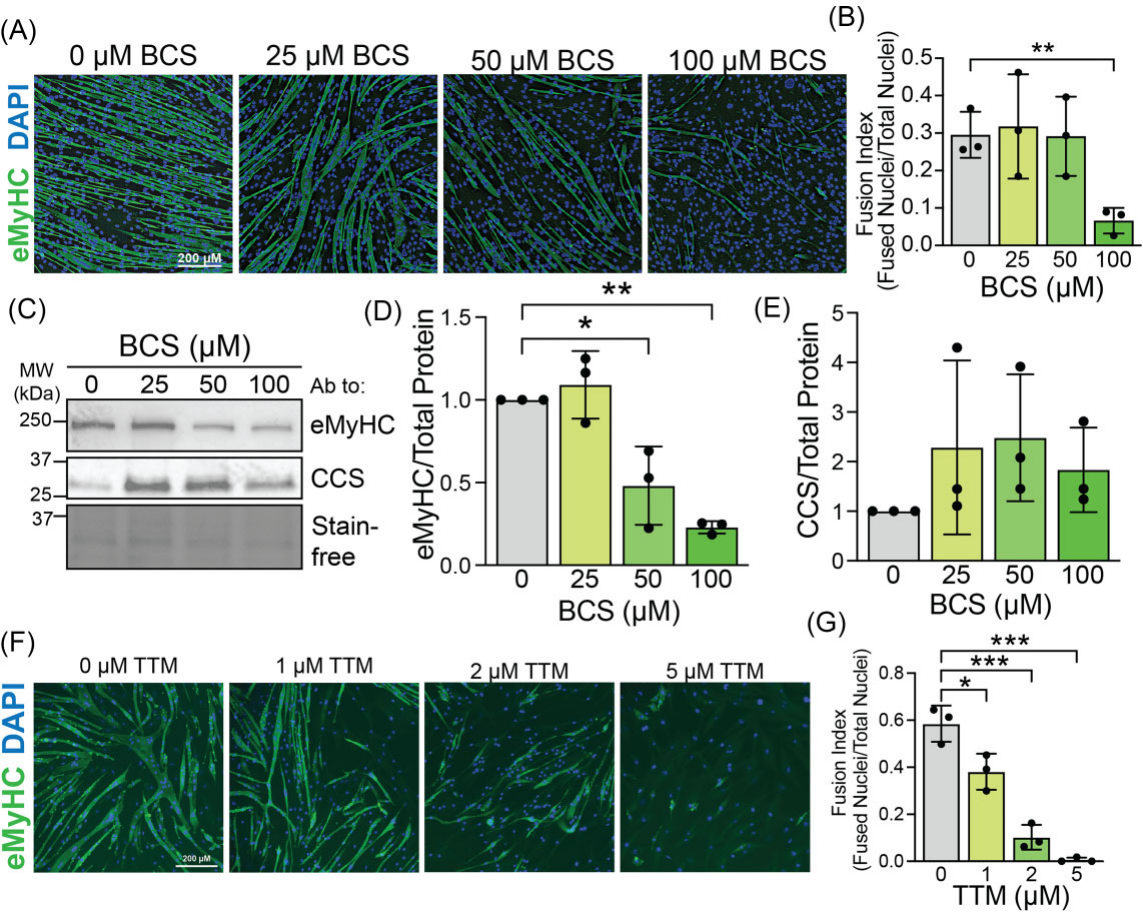
**Extracellular and intracellular Cu chelators impair C2C12 differentiation.** (A) C2C12 myotubes induced to differentiate in the presence of 0, 25, 50, or 100 µM bathocuproine disulfonic acid (BCS) stained with an antibody to embryonic myosin heavy chain (eMyHC). Nuclei are stained with DAPI. Bar = 200 µm. (B) Fusion index (fraction of fused nuclei per total nuclei) calculated for cells treated with BCS and induced to differentiate showing a significantly lower fusion index in cells treated with 100 µM BCS. (C) Immunoblot of lysates from differentiating BCS-treated C2C12 cells probed with antibodies to eMyHC and the Cu chaperone for SOD1 (CCS). Total protein is shown using Stain-free gel imaging technology. Quantification for eMyHC (D) and CCS (E) as measured by densitometry and normalized to total protein showing significant decrease in eMyHC levels in BCS-treated samples and trend toward increased CCS levels in samples treated with 100 µM BCS. (F) C2C12 myotubes induced to differentiate in the presence of 0, 1, 2, or 5 µM tetrathiomolybdate (TTM) stained with an antibody to eMyHC. Nuclei are stained with DAPI. Bar = 200 µm. (G) Fusion index (fraction of fused nuclei per total nuclei) calculated for cells treated with TTM and induced to differentiate revealing a significant dose-dependent decrease in fusion index with TTM treatment. Shown is the mean ± standard deviation for *n* = 3 experiments. For all experiments, statistical significance was determined using one-way ANOVA (*P < 0.05, **P < 0.01, ***P < 0.001).

Intracellular Cu levels increase early in differentiation of both primary and C2C12 myoblasts.^26^ Considering that serum contains potential Cu coordinating ligands like albumin and amino acids, the elevated Cu associated with differentiation could be a result of increased availability of Cu in the low serum Dulbecco's modified Eagle medium (DMEM) used to differentiate C2C12 cells. However, addition of Cu binding molecules including bovine serum albumin (BSA) or histidine (His) did not inhibit Cu hyperaccumulation in differentiating myoblasts (Fig. 1A). Calcium (Ca) levels were used as an internal control as Ca is expected to increase during differentiation (Fig. 1B). These data suggest that elevated Cu levels in differentiating myoblasts are not attributable to serum deprivation. Our previous study showed that addition of tetraethylenepentamine (TEPA) inhibits differentiation of primary myoblasts.^26^ Since TEPA may also bind zinc, the extracellular Cu-specific chelator bathocuproine disulfonic acid (BCS) was added to differentiating myoblasts to confirm that Cu is required for differentiation. Immunostaining with an antibody to embryonic myosin heavy chain (eMyHC) was used to visualize myotubes (Fig. 2A). Addition of 100 µM BCS led to a decrease in myotube formation as quantified by fusion index (nuclei in myotubes/total nuclei) (Fig. 2B). Immunoblots for markers of differentiation (Fig. 2C) revealed a significant decrease in the myogenic marker eMyHC (Fig. 2D). Levels of CCS are inversely correlated to intracellular Cu levels and are thus used to indicate Cu availability.^30,31^ Myoblasts differentiated in the presence of BCS had increased levels of CCS, indicating that BCS reduced Cu availability (Fig. 2E). To confirm that intracellular Cu is required for differentiation, myoblast were induced to differentiate in the presence of the strong intracellular chelator tetrathiomolybdate (TTM), which can strip Cu from intracellular proteins. TTM impaired differentiation in a dose-dependent manner with 5 µM TTM completely inhibiting myotube formation (Fig. 2F, G). Similarly to BCS, the presence of TTM led to an increase in CCS levels and decreases in markers for myogenesis (Supplementary Fig. S1). These results demonstrate that both Cu influx and intracellular Cu are required for differentiation of C2C12 myoblasts.

**Fig. 3:**
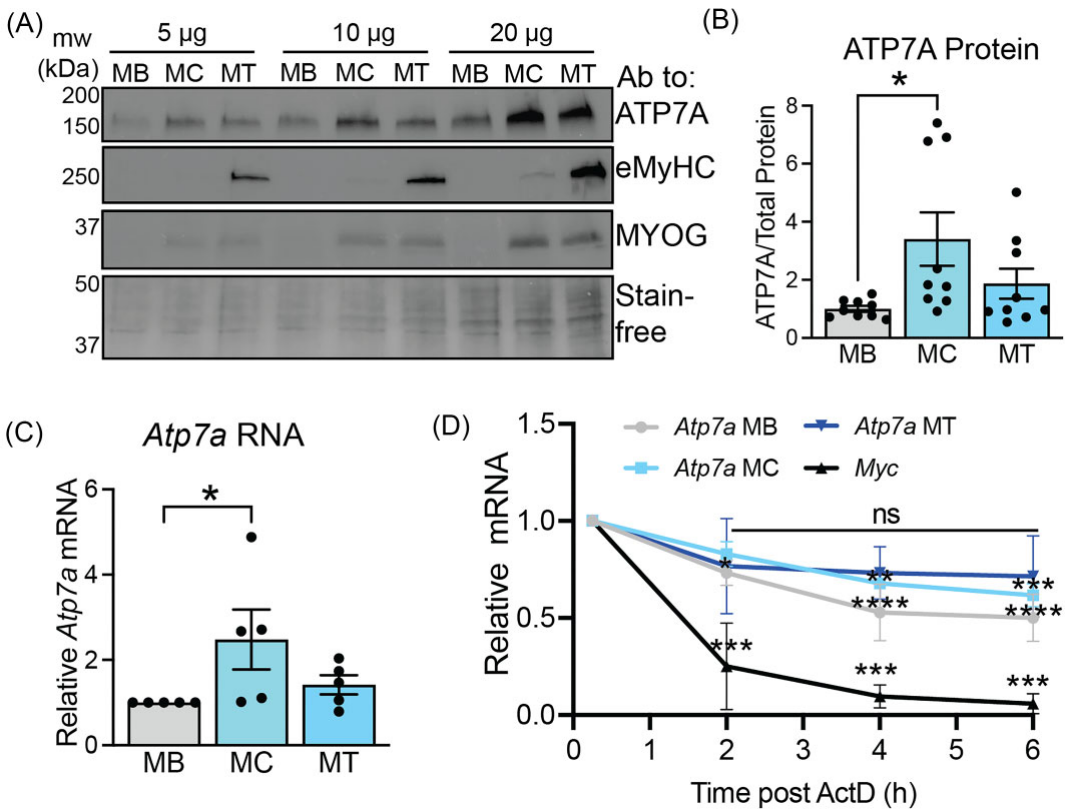
ATP7A levels and Atp7a RNA stability correlate fluctuate during C2C12 differentiation. (A) Immunoblot of lysate from myoblasts (MB), myocytes (MC), and myotubes (MT) probed with antibodies to ATP7A, eMyHC, and MYOG. Total protein is shown using Stain-free gel technology. Shown is a representative image of *n* = 1 loaded at 5, 10, and 20 µg per well. (B) Quantification of ATP7A protein as measured by densitometry and normalized to total protein showing significant increase in ATP7A in MC. Shown is mean ± standard deviation for *n* = 9. (C) Steady-state levels of Atp7a RNA showing increase in Atp7a RNA in MC as measured by qRT-PCR and normalized to Rplp0. Shown is mean ± standard deviation for *n* = 6 experiments. (D) Actinomycin D chase assays measuring Atp7a RNA stability. In MB, MC, and MT. Myc was used as a control. Shown is mean ± standard deviation for *n* = 3 experiments. Statistical significance was determined using one-way ANOVA (*P < 0.05, **P < 0.01, ***P < 0.001, ****P < 0.0001).

In primary myoblasts, ATP7A is known to increase in differentiated myotubes relative to myoblasts.^26^ Here, immunoblots were used to probe lysates from differentiating C2C12 cells with antibodies to ATP7A, myogenin (an early differentiation marker activated in myocytes), and eMyHC (a marker for mature myotubes) (Fig. 3A). Increased levels of ATP7A were detected in the myocyte/early myotube stage, when myogenin but not eMyHC is expressed (Fig. 3B). Steady-state *Atp7a* RNA levels were significantly increased in the myocyte/early myotube stage (Fig. 3C). Using actinomycin D chase assays, we found that the *Atp7a* RNA was more unstable in myoblasts (*t*_1/2_ ∼ 5 h) compared to myocytes (*t*_1/2_ ∼ 8 h) as measured by significant decrease in *Atp7a* levels over 6 h of actinomycin D treatment and that no significant decrease was detected in fully mature myotubes (*t*_1/2_ ∼ 9 h) (Fig. 2D, Supplementary Table S1) These data suggest that the *Atp7a* transcript is stabilized during the maturation of myotubes and likely contributes to the increased ATP7A protein levels. Since myogenesis is associated with elevated Cu accumulation (Fig. 1A), we tested the effect of Cu on *Atp7a* RNA stability during myogenesis. C2C12 myoblasts were grown in 20 µM CuSO_4_ or 20 µM TEPA for 4 d to raise or lower intracellular Cu, respectively (Supplementary Fig. S2A). *Atp7a* mRNA in myoblasts grown in Cu (*t*_1/2_ ∼ 19 h) was stabilized relative to *Atp7a* mRNA in untreated myoblasts (Supplementary Fig. S2B; Supplementary Table S1). Interestingly, limiting Cu import by growing myoblasts in the presence extracellular Cu chelator TEPA decreased did not affect *Atp7a* RNA stability (*t*_1/2_ ∼ 6 h), suggesting that stabilization of *Atp7a* mRNA upon differentiation to myotubes may be driven by increased Cu.

### The *Atp7a* RNA 3′ UTR negatively regulates RNA in myoblasts but not myotubes

**Fig. 4:**
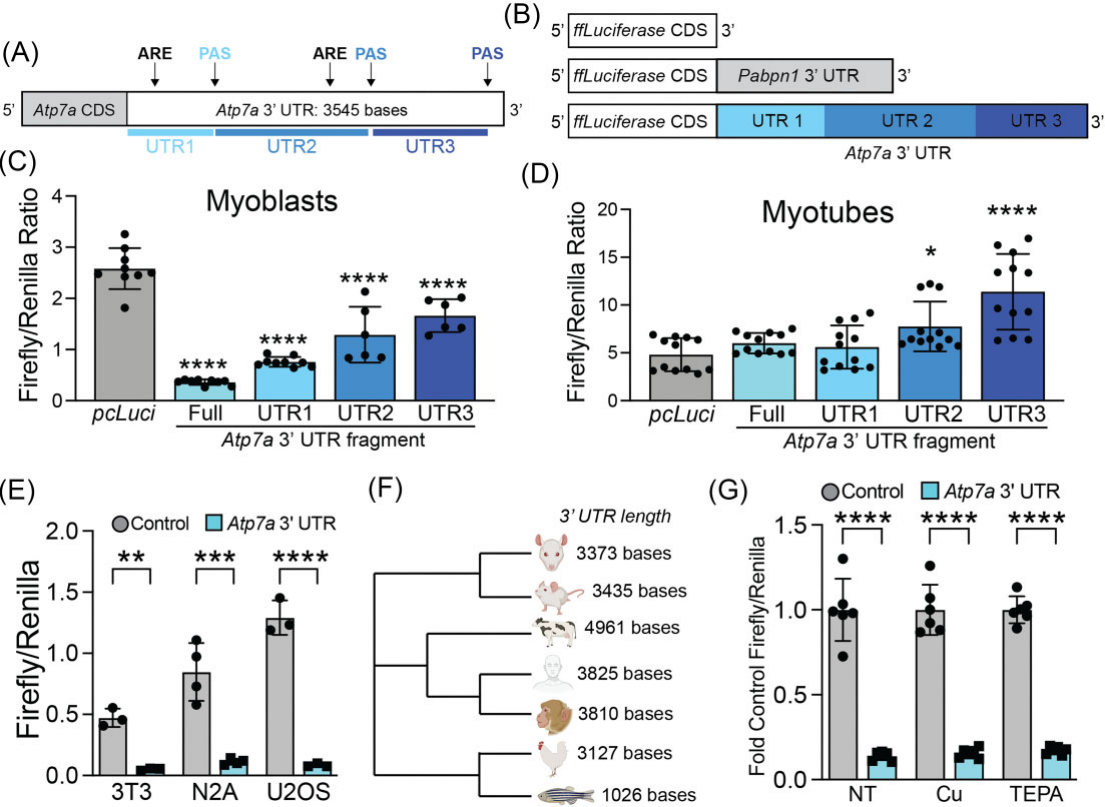
The Atp7a RNA 3′ UTR imparts differentiation-dependent regulation. (A) Schematic of the fulllength 3545 base Atp7a 3′ UTR showing polyadenylation signals (PAS) and AU-rich elements (ARE). The truncated UTR fragments are shown in shades of blue. (B) Schematic of reporter constructs containing the firefly luciferase gene alone or the firefly luciferase gene in the presence of the 3′ UTR from Pabpn1 or Atp7a with truncated UTR fragments shown in blue. (C) Luciferase activity assays in measured as firefly/Renilla luciferase activity in C2C12 myoblasts showing significant decreases in the presence of the full-length Atp7a 3′ UTR and all fragments with the strongest effect in full-length and fragment 1. Shown is mean ± standard deviation for *n* = 6–9 experiments. (D) Luciferase activity assays in C2C12 myotubes showing of the fulllength Atp7a 3′ UTR or the UTR1 fragment and significant increase in luciferase activity in the presence of UTR fragments 2 and 3. Shown is mean ± standard deviation for *n* = 12 experiments. (E) Luciferase activity assays in proliferating 3T3, N2A, and U2OS cells showing similar significant decrease in firefly/Renilla luciferase activity in the presence of the full-length Atp7a 3′ UTR. Shown is mean ± standard deviation for *n* = 3–4 experiments. (F) Neighbor-joining phylogenetic tree of 3′ UTR sequences from reported or predicted RefSeq genes for multiple vertebrate species. (G) Luciferase activity assays in C2C12 myoblasts grown in the presence of 20 µM CuSO4 or 20 µM TEPA showing no effect of Cu or TEPA on firefly/Renilla luciferase activity. Shown is mean ± standard deviation for *n* = 6 experiments. Statistical significance was determined using one-way ANOVA except in L, which was determined using two-way ANOVA (*P < 0.05, **P < 0.01, ***P < 0.001, ****P < 0.0001).

To identify elements involved in *Atp7a* RNA turnover, we looked to the 3′ UTR as a potential regulatory control region. The murine *Atp7a* 3′ UTR is 3545 bases long and contains multiple conserved regulatory elements including polyadenylation signals (PAS) and cleavage signals, adenosine and uridine (AU)-rich elements (ARE), and guanosine and uridine (GU)-rich elements (GRE) (Fig. 4A). To determine if the 3′ UTR contributes to differentiation-dependent regulation of *Atp7a*, the full-length *Atp7a* 3′ UTR sequence was inserted downstream of a firefly luciferase gene in a pcDNA 3.1 mammalian expression plasmid (Fig. 4B). A similar construct containing the *Pabpn1* 3′ UTR, which does not impart negative regulation in myoblasts,^32^ was used as a control. The presence of the *Atp7a* 3′ UTR significantly decreased luciferase activity by ∼8.5-fold in transfected myoblasts compared to controls, an effect that was significantly blunted in myotubes (Fig. 4C). This result indicates that the *Atp7a* 3′ UTR can function as an autonomous suppressor of mRNA stability in myoblasts and is derepressed in response to elevated Cu levels upon differentiation into myotubes.

During primary myoblast differentiation, the PAS in the *Atp7a* 3′ UTR shifts from a distal to a proximal PAS.^26^ To alternative PAS utilization contributes to myoblast-specific negative regulation of the luciferase reporter, the *Atp7a* 3′ UTR was truncated to the first 1145 bases at the proximal PAS (Fig. 4B). However, there was only a very minor effect of this deletion on luciferase activity as compared to the full-length *Atp7a* 3′ UTR (Fig. 4D), suggesting that the negative regulation stems primarily from the proximal portion of *Atp7a* 3′ UTR. To determine if the *Atp7a* 3′ UTR functions autonomously in other proliferating cell types, N2A neuroblastoma cells and NIH 3T3 fibroblasts were transfected with luciferase reporters containing the full-length *Atp7a* 3′ UTR. In both cell types, the presence of the *Atp7a* 3′ UTR significantly decreased luciferase activity (Fig. 4E). This result suggests that the negative regulation imparted by the *Atp7a* 3′ UTR region is not unique to myoblasts. To determine if Cu influences regulation by the *Atp7a* 3′ UTR, we assayed luciferase activity in transfected cells treated with a physiological dose of Cu (20 µM) or the chelator TEPA (20 µM). No difference in luciferase activity was detected in either Cu- or TEPA-treated samples (Fig. 4F), suggesting that Cu alone is not sufficient to promote *Atp7a* stabilization via the 3′ UTR in myoblasts. These results indicate that the proximal portion of the *Atp7a* 3′ UTR mediates negative regulation in proliferating cells and, in the context of C2C12 myoblasts, this regulation is relieved upon differentiation.

### ATP7A is required for C2C12 myoblast differentiation

**Fig. 5:**
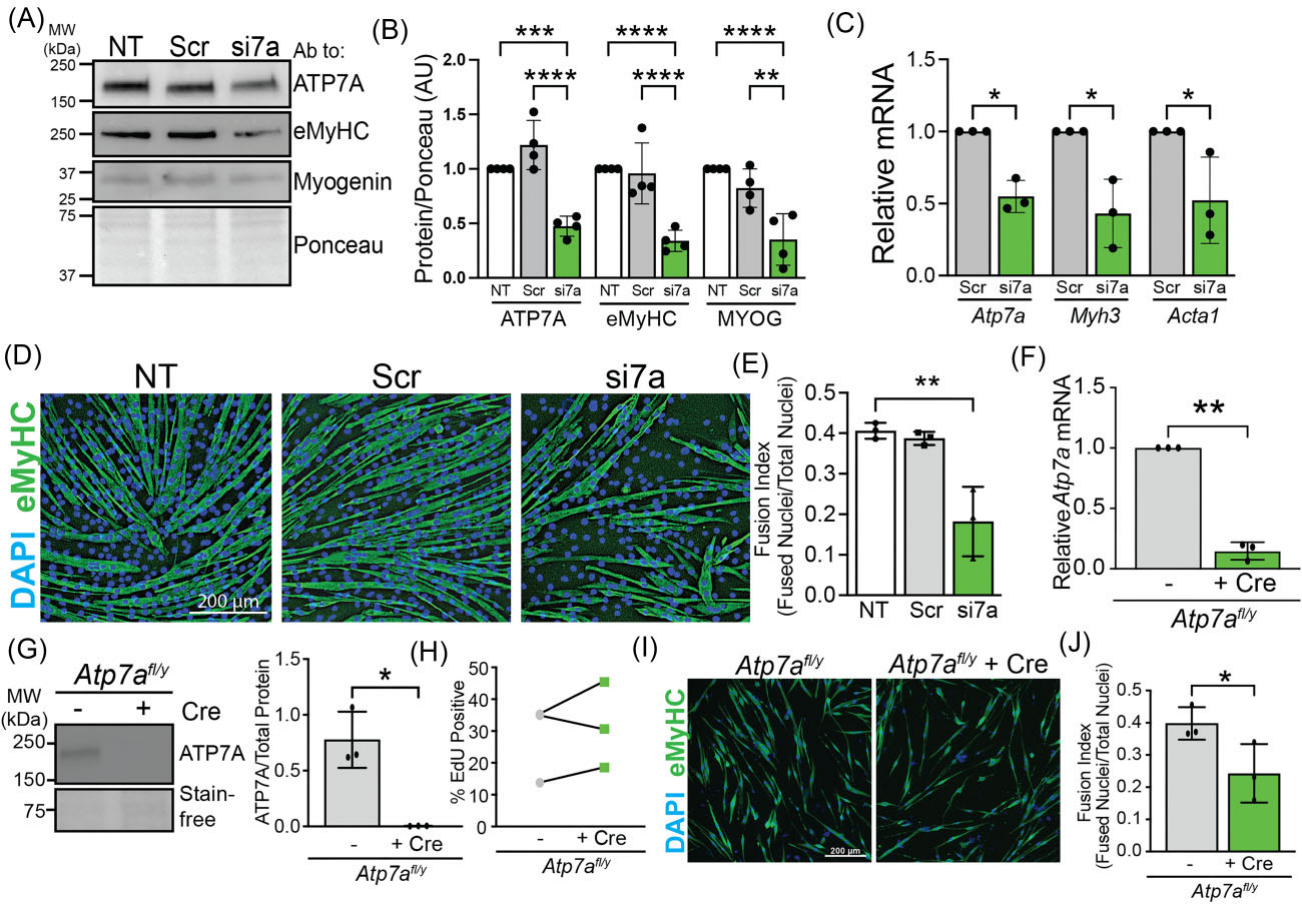
ATP7A is required for C2C12 and primary myoblast differentiation. (A) Immunoblot of myotube lysates from nontreated control (NT), nontargeting siRNA (Scr), and Atp7a knockdown (si7a) probed with antibodies to ATP7A, eMyHC, myogenin, and with total protein shown using Ponceau stain. (B) Quantification of ATP7A by densitometry as normalized to total protein showing a ∼50% knockdown of ATP7A protein in si7A sample and a significant decrease in eMyHC and myogenin (MYOG) in si7a samples. (C) RT-qPCR showing a ∼50% Atp7a knockdown in si7a samples and significant decreases in myogenic markers Myh3 (encoding eMyHC) and Acta1. (D) NT, Scr, or si7a C2C12 myotubes induced to differentiate and stained with an antibody to eMyHC. Nuclei are stained with DAPI. Bar = 200 µm. (E) Fusion index calculated from images shown in D revealing a ∼50% decrease in si7a samples. (F) Steady-state Atp7a RNA levels in primary myoblasts isolated from Atp7afl/y mice with (+ Cre) or without (-) Cre-mediated recombination showing a significant decrease in Atp7a RNA after Cre treatment. (G) Immunoblot of lysates from primary myoblasts isolated from Atp7afl/y mice with or without Cre-mediated recombination probed with an antibody to ATP7A and total protein imaged using Stain-free gel technology. (H) Quantification of blot shown in G revealing no detectable ATP7A protein after Cre treatment. (I) Atp7afl/y myoblasts with and without Cre were induced to differentiate and stained with an antibody to eMyHC and DAPI to visualize nuclei. Bar = 200 µm. (J) Fusion index calculation revealing a ∼50% decrease in myotube formation in Atp7afl/y + Cre samples. (K) Proliferation of Atp7afl/y myoblasts with and without Cre as measured by EdU labeling showing no decrease in percentage EdU positive cells after Cre treatment. Shown is mean ± standard deviation for *n* = 3–4 experiments. For (B), (C), and (E), statistical significance was determined using one-way ANOVA and for (F), (H), (J), and (K) statistical significance was determined by *t*-test (*P < 0.05, **P < 0.01).

To determine if ATP7A is required for muscle cell differentiation, siRNA was used to knock down *Atp7a* in C2C12 cells resulting in approximately 50% loss of ATP7A (Fig. 5A, B). Silencing of ATP7A impaired differentiation of C2C12 cells as detected by a significant decrease in the levels of total eMyHC protein (Fig. 5C) and by a significant decrease in myotube formation (Fig. 5D, E). To confirm the ATP7A requirement for skeletal muscle cell differentiation, we isolated primary myoblasts from male mice containing a floxed *Atp7a* allele (*Atp7a^fl/y^*) and transduced them with adenoviral-encoded Cre recombinase. Transduction with adenoviral Cre led to a significant decrease in *Atp7a* RNA (Fig. 5F) and loss of detectable ATP7A protein (Fig. 5G, H). Control- and Cre-treated *Atp7a^fl/y^* myoblasts were plated for differentiation and after approximately 72 h the fusion index was measured. Immunofluorescence staining for eMyHC and fusion index quantification revealed that myotube formation was impaired in Cre-treated *Atp7a^fl/y^* myoblasts (Fig. 5I, J). These data suggest that ATP7A is required for differentiation of primary myoblasts *in vitro*. This differentiation defect was not due to impaired proliferation as equal numbers of cells were plated for differentiation assays and no proliferation defect was detected by ethynyl-2′-deoxyuridine (EdU) labeling (Fig. 5K). Taken together, these data suggest that ATP7A is essential for differentiation of both C2C12 and primary myoblasts.

The Cu chaperone ATOX1 delivers Cu to ATP7A and ATP7B.^33^ Steady-state levels of ATOX1 protein increased nearly threefold in differentiated C2C12 myotubes compared to myoblasts (Supplementary Fig. S3A, B). However, *Atox1* knockdown did not impair differentiation as measured by eMyHC or Myogenin levels (Supplementary Fig. S3C, D). Although there appeared to be fewer myotubes in *Atox1* knockdowns (Supplementary Fig. S3E), there was no significant effect on fusion index (Supplementary Fig. S3F). This result supports a model where ATP7A can acquire Cu in the absence of ATOX1 and is consistent with the milder phenotype of *Atox1* vs. *Atp7a* knockout mice.^34^ The related Cu transporter ATP7B was not detected by immunoblot or qRT-PCR (Supplementary Fig. S3G), and is thus unlikely to function in C2C12 differentiation.

**Fig. 6:**
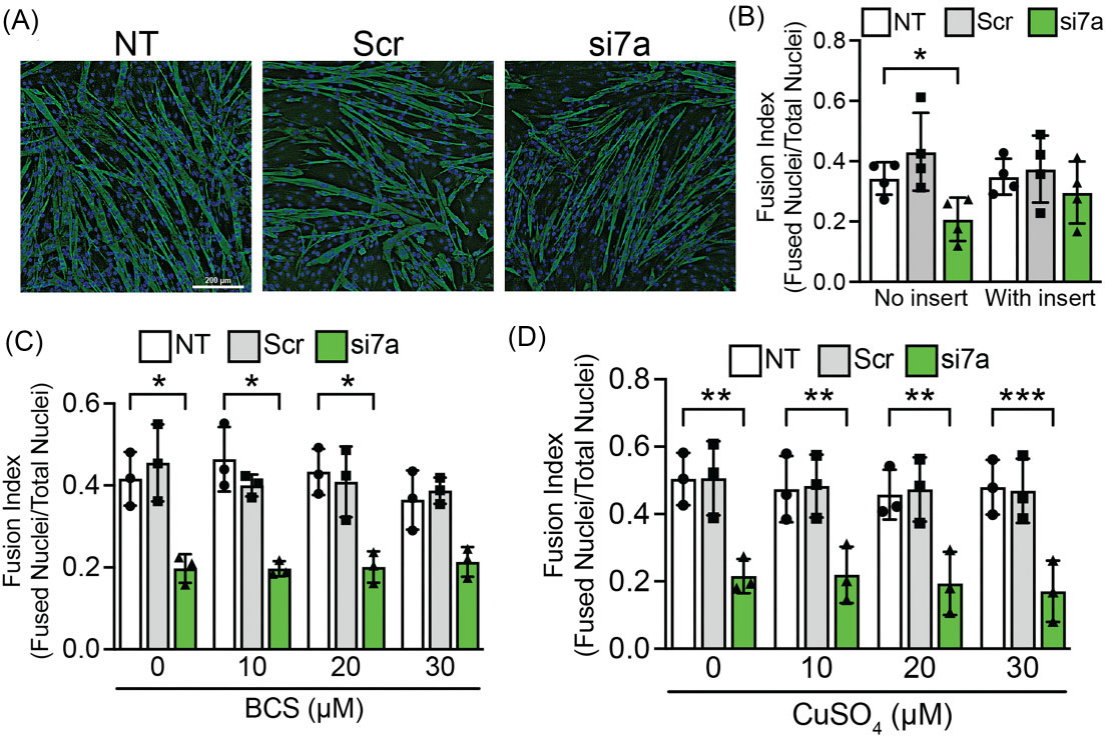
ATP7A provides a secreted factor to promote C2C12 differentiation. (A) Nontreated control (NT), nontargeting siRNA (Scr), and Atp7a knockdown (si7a) cells were induced to differentiate in the presence of trans-well inserts containing control cells and stained with an antibody to eMyHC and DAPI to visualize nuclei. Bar = 200 µm. (B) Quantification of fusion index for trans-well experiments revealing a significant decrease in myotube formation in si7a samples with no insert but no significant effect on myotube formation in si7a samples with trans-well inserts containing control cells. (C) Fusion index calculations for Atp7a knockdown cells (si7a) treated with 0, 10, 20, and 30 µM BCS showing a significant decrease in myotube formation in si7a samples relative to control at all BCS concentrations. (D) Fusion index calculations for Atp7a knockdown cells (si7a) treated with 0, 10, 20, and 30 µM CuSO4 showing a significant decrease in myotube formation in si7a samples relative to control at all CuSO4 concentrations. Shown is mean ± standard deviation for *n* = 3–4 experiments. Statistical significance was determined using one-way ANOVA (*P < 0.05, **P < 0.01, ***P < 0.001).

A major function of ATP7A is to deliver Cu into the secretory pathway to metallate secreted cuproenzymes, so we initiated a series of experiments aimed at testing whether the loss of secreted cuproenzyme activity may underlie the differentiation defect caused by ATP7A silencing in C2C12 cells. To this end, we tested whether wild-type C2C12 cells cultured in trans-wells can rescue the differentiation defect in ATP7A-silenced cells. Differentiating wild-type cells in trans-wells with 0.4 µm pores, which allows passage of proteins but not cells, was sufficient to rescue myotube formation in *Atp7a* knockdown cells (Fig. 6A, B). Adding 0–30 µM BCS, which is well below concentrations used to inhibit differentiation (Fig. 2B), did not rescue myotube formation (Fig. 6C), suggesting that impaired differentiation of *Atp7a* deficient myoblasts is not caused by Cu toxicity. Furthermore, the addition of exogenous CuSO_4_ did not rescue the differentiation defect detected in *Atp7a* knockdown cells (Fig. 6D), suggesting that the requirement for ATP7A cannot be bypassed by the addition of Cu salts. Taken together, these results suggest that Cu transport to secreted Cu-dependent enzymes is a critical function of ATP7A during myogenesis.

### ATP7A is required to deliver Cu to LOX during C2C12 differentiation

**Fig. 7:**
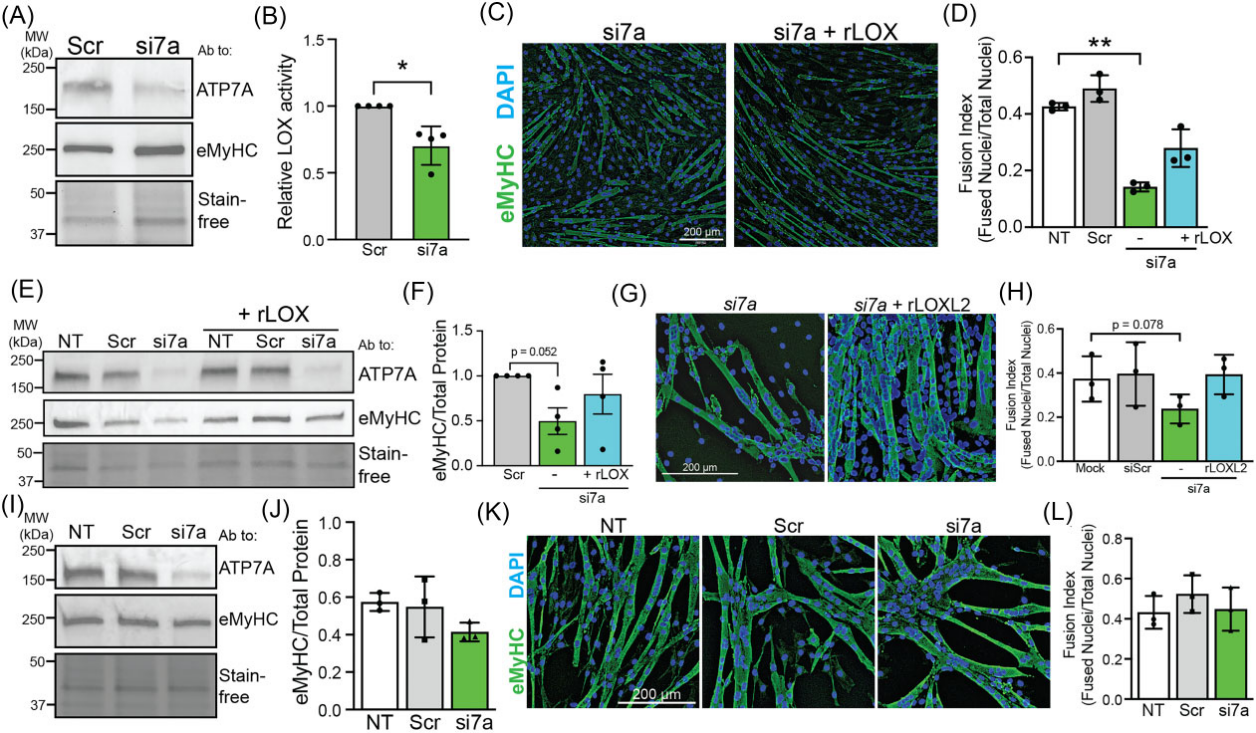
ATP7A is required to provide Cu to LOX for C2C12 myoblast differentiation. (A) Immunoblot of lysates from myotubes treated with nontargeting negative control (Scr) or Atp7a siRNA (si7a) probed with antibodies to ATP7A, eMyHC, and total protein imaged with Stain-free technology. (B) Quantification of relative LOX activity showing a significant decrease in LOX activity in si7a myotubes relative to Scr control myotubes. (C) Atp7a knockdown cells were induced to differentiate with (si7a + LOX) and without (si7a) added rLOX and stained with an antibody to eMyHC. Bar = 200 µm. (D) Quantification of fusion index reveals a significant decrease in myotube formation in si7a cells relative to nontreated control (NT) but no significant defect in si7a cells treated with rLOX. (E) Immunoblot of lysates from NT, Scr, and siRNA cells differentiated with and without added rLOX probed with antibodies to ATP7A, eMyHC, and total protein shown using Stain-free imaging. (F) Quantification of eMyHC protein revealing a decrease in si7a cells that is not detected in si7a cells with added rLOX. (G) Atp7a knockdown cells were induced to differentiate with (si7a + rLOXL2) and without (si7a) added rLOXL2 and stained with an antibody to eMyHC. Bar = 200 µm. (H) Quantification of fusion index reveals a significant decrease in myotube formation in si7a cells relative to nontreated control (NT) but no defect in si7a cells treated with rLOXL2. (I) Immunoblot of lysates from NT, Scr, and si7a cells plated on Matrigel-coated plates prior to inducing differentiation probed with antibodies to ATP7A, eMyHC, and total protein shown with Stain-free imaging. (J) Quantification of blot showing no change in eMyHC. (K) NT, Scr, and si7a cells were plated on Matrigel prior to differentiation, induced to differentiate, and then stained with an antibody to eMyHC and DAPI to visualize nuclei. Bar = 200 µm. (L) Fusion index for NT, Scr, and si7a plated on Matrigel revealing no defect associated with Atp7a knockdown. Shown is mean ± standard deviation for *n* = 3–4 experiments. For (B), statistical significance was determined by *t*-test and for (D), (F), (H), (J), and (L), statistical significance was determined using one-way ANOVA (*P < 0.05, **P < 0.01).

ATP7A delivers Cu to a variety of Cu enzymes in the secretory pathway. Several secreted cuproenzymes, including LOX, Cu-dependent amine oxidase (AOC3), and extracellular superoxide dismutase (SOD3) are expressed in muscle. Of these, only LOX is known to be required for myoblast differentiation.^28,29^ To determine whether ATP7A facilitates myoblast differentiation via LOX, we first measured LOX levels and activity in conditioned medium from myoblasts and myotubes. LOX protein was undetectable in conditioned medium from myoblasts but was abundant in conditioned medium from myocytes and eMTs (Supplementary Fig. S4A), which is similar to the timing at which ATP7A protein levels increase (Fig. 3A, B). LOX activity was elevated in the conditioned medium from myotubes relative to myoblasts (Supplementary Fig. S4B) and the LOX inhibitor β-aminopropionitrile (βAPN) impaired differentiation of C2C12 myoblasts to myotubes (Supplementary Fig. S4C, D). To test the ATP7A dependence of LOX activity, *Atp7a* was knocked down in the early myotube stage to prevent the confounding effects of impaired differentiation caused by ATP7A deficiency. Knocking down *Atp7a* in early myotube did not impair differentiation, as no change in eMyHC levels was detected (Fig. 7A). As expected, a small but significant decrease in LOX activity was detected upon *Atp7a* knockdown (Fig. 7B). Overall, these data indicate that ATP7A delivers Cu to LOX, which is required for C2C12 myoblast differentiation.

To confirm the importance of the Cu–ATP7A–LOX axis in C2C12 differentiation, we tested whether exogenous LOX added to the media of ATP7A deficient cells could restore their ability to differentiate. We first performed *in vitro* LOX activity assays on commercial recombinant human LOX (rLOX). Surprisingly, rLOX showed very little activity relative to rLOX homolog 2 (rLOXL2), which was used as a positive control (Supplementary Fig. S4E). However, adding rLOX to the differentiation medium in *Atp7a* knockdown cells resulted in an increase in total LOX activity (Supplementary Fig. S4F). LOX is secreted as a zymogen and cleaved by BMP-1, Tolloid like-1, and other proteases to generate mature active LOX.^35^ Thus, commercially available rLOX may not be fully processed until it is added to conditioned medium containing the necessary proteases. After adding rLOX to differentiating *Atp7a* knockdown cells, we detected a partial rescue of myotube formation (Fig. 7C, D) as well as partial restoration of total eMyHC levels (Fig. 7E, F). To confirm that LOX activity can restore myotube formation in *Atp7a* knockdown cells, we tested rLOXL2, which exhibited strong activity *in vitro* (Supplementary Fig. S4E). As expected, rLOXL2 restored myotube formation in *Atp7a* knockdown cells to wild-type levels (Fig. 7G, H). Unlike LOX, proteolytic cleavage of LOXL2 is not necessary for activity,^36^ which may account for the stronger rescue by rLOXL2. Since LOX activity is involved in modifying the extracellular matrix, we considered the effect of providing additional matrix to ATP7A deficient cells. When differentiated on Matrigel, the differentiation defect in *Atp7a* knockdown myoblasts was rescued as determined by restoration of eMyHC levels to near wild-type levels (Fig. 7I, J) and fusion index (Fig. 7K, L). However, plating on Matrigel caused ATP7A deficient myoblasts to form large, globular multinucleated cells rather than elongated myotubes (Fig. 7K, Supplementary Fig. S4F) suggesting aberrant migration and/or fusion. Taken together, these experiments strongly suggest that ATP7A is needed to deliver Cu to LOX for normal myotube formation during C2C12 myoblast differentiation.

## Discussion

In this study, we sought to understand how regulated expression of Cu transporters contributes to differentiation-dependent Cu requirements. We found that C2C12 skeletal myoblasts require an influx of Cu during differentiation into myotubes, which is associated with increased levels of the trans-Golgi Cu transporter ATP7A. Our data suggest that the *Atp7a* mRNA increases during differentiation, which is mediated in part by derepression of its turnover. The *Atp7a* 3′ UTR contains elements that impart negative regulation of luciferase reporters in myoblasts and other proliferating cells but not in myotubes. The finding that ATP7A levels increase in myocytes and early myotube prompted us to test the requirement for ATP7A during differentiation. Using independent methods of ATP7A silencing in C2C12 (siRNA) and primary myoblasts (Cre recombinase) we detected impaired myotube formation caused by ATP7A deficiency. Myotube formation was rescued in trans by the conditioned medium of wild-type cells, suggesting that a secreted cuproprotein may underlie this defect. Candidates included the LOX family of enzymes since the prototypic member, LOX, is required for *in vitro* and *in vivo* myogenesis.^28,29^ Supplementation of exogenous LOX or LOXL2 to the medium restored differentiation in ATP7A-deficient myoblasts. Taken together, these results suggest that ATP7A expression is stabilized early during myoblast differentiation in order to provide adequate Cu to LOX enzymes needed for myotube formation.

**Fig. 8:**
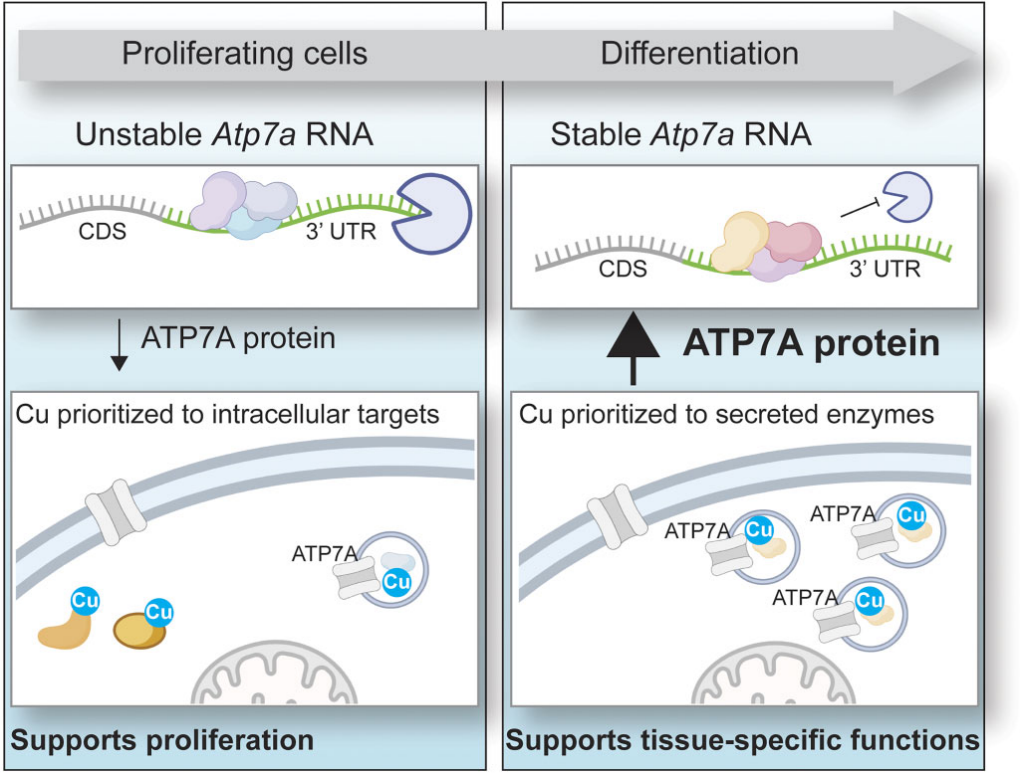
**Overall model** The data reported here lead to a working model wherein Atp7a RNA in proliferating cells is destabilized via the 3′ UTR to prioritize limited Cu to intracellular Cu binding proteins that promote proliferation. In differentiating cells, Atp7a RNA is stabilized to allow Cu to flow to ATP7A for delivery to secreted cuproenzymes required for differentiation.

The major proteins involved in Cu homeostasis in mammalian cells have been identified, but the mechanisms by which these proteins respond to changing Cu demands in a tissue-specific or temporal fashion are not well understood. Expression of LOX increases dramatically during myoblast differentiation and genetic studies have shown that LOX is essential for developmental and regenerative muscle differentiation.^28,29^ Our data suggest that regulatory control of LOX-mediated myoblast differentiation occurs not only via increased LOX expression but also through increased expression of ATP7A mediated by message stabilization. As such, the data point to the following model of muscle differentiation outlined in Fig. 8. Proliferating myoblasts are low in Cu due to low levels of CTR1 and express very little ATP7A and LOX. Upon differentiation to myocytes and myotubes, an as-yet unidentified stimulus leads to increased CTR1 expression and Cu uptake, which facilitates Cu-dependent stabilization of *Atp7a* RNA. The resulting increase in ATP7A expression drives more Cu into the secretory pathway to LOX, a protein required for myoblast differentiation. Thus, the increased Cu demand for LOX biosynthesis in differentiating cells appears to be regulated at multiple levels.

Several studies have pointed toward the relevance of post-transcriptional control of Cu in eukaryotes. In *Saccharomyces cerevisiae*, nonsense mediated decay controls expression of RNAs encoding the Cu transporter Ctr2 and the metallochaperones Cox17, Cox19, and Cox23.^37,38^ RNA binding proteins and microRNAs contribute to cisplatin resistance by regulating *ATP7A* and *CTR1* in several cancer cell lines, suggesting that post-transcriptional regulation may be a widely used phenomenon to control Cu distribution.^39–42^ Furthermore, gene ontology analysis revealed that siRNAs targeting RNA splicing and RNA localization biological processes were enriched in HeLa cells with altered Cu levels in a screen for regulators of trace element homeostasis.^43^ More recently, McCann and colleagues reported that the RNA binding protein HnRNP A2/B1 regulates *ATP7A* RNA via the 3′ UTR during neuronal cell differentiation in an isoform specific manner.^44^ Additionally, this study identified an HnRNP A2/B1 binding site in the proximal *ATP7A* 3′ UTR, which is in agreement with our finding that the proximal *Atp7a* 3′ UTR imparts negative regulation. Taken together, these studies suggest that regulation of *ATP7A* RNA via the 3′ UTR plays an important role in mammalian cell differentiation.

It is likely that regulation of *ATP7A* involves the coordination of multiple proteins bound to the 3′ UTR and other regions of the RNA. Several RNA binding proteins are important in skeletal muscle development, regeneration, and function.^45–48^ The previously identified *ATP7A* regulator, HNRNPA2/B1 is required for myoblast differentiation and levels increase in regenerating muscle and during *in vitro* myogenesis.^45^ HNRNPA2/B1 deficiency impairs myogenesis *in vitro*^45^ and frameshift variants cause an early onset oculopharyngeal muscular dystrophy-like disease.^49^ Other RNA binding proteins have been predicted to interact with the proximal portion of the *ATP7A* 3′ UTR including HuR, HNRNPA1, HNRNPH1, ALY, RBFOX1, PTBP1, and HNRNPC (Supplementary Table S2).^50,51^ Of these, several are important for skeletal muscle development, regeneration, and function. HuR binds AREs and promotes myoblast differentiation and resistance to cachexia in mature muscles. Conversely, PTBP1 inhibits myogenesis and levels decrease as myoblasts differentiate to myotubes. Future studies are needed to elucidate the role of HNRNPA2/B1 and other RNA binding proteins in modulating ATP7A levels in skeletal muscle and other tissues.

Similar to differentiation-dependent *Atp7a* RNA stabilization, we detected a Cu-dependent increase in *Atp7a* RNA stability in myoblasts that was not detected in luciferase reporters, suggesting that additional regulatory mechanisms may contribute to Cu-dependent regulation of ATP7A. Future studies to identify RNA binding proteins, microRNAs, and RNA decay pathways that control ATP7A will provide a clearer picture of how Cu distribution is regulated post-transcriptionally and how this mode of regulation integrates with transcriptional and post-translational control of Cu transporters.

Our data showing that ATP7A is required for muscle cell differentiation is consistent with an emerging model wherein ATP7A and Cu delivery to secreted cuproproteins are an integral component of cellular differentiation and tissue development.^13^ In adipocyte maturation, ATP7A levels and Cu flux to the secretory pathway increase in conjunction with increased expression of the AOC3, which governs metabolic fuel selection.^52^ Similarly, during neuronal differentiation, increasing levels of Cu and ATP7A correlates with the expression of neuronal subtype specific cuproenzymes like peptidyl-glycine-α-monooxygenase (PAM) and dopamine-β-hydroxylase.^53^ Interestingly, though Cu flux in neuronal differentiation appears to depend in part on the presence of the Cu chaperone ATOX1, we found that muscle cell differentiation does not require ATOX1. This result suggests that alternative pathways exist for Cu delivery to ATP7A in muscle.

In the case of ATP7A deficiency, the rescue of impaired differentiation by exogenous LOX or LOXL2 suggests that these defects are attributable to the lack of ATP7A-dependent metalation of LOX/LOXL proteins required for topoquinone cofactor biosynthesis^54^ in the secretory pathway. LOX is important for fetal/neonatal and regenerative myogenesis due to its function in cross-linking collagen and modifying cellular signaling molecules such as the TGF-β1 signaling ligand, though it may also have an intracellular function in modifying the transcription factor VGLL3.^28,29^ LOX also influences activation of focal adhesion kinase (FAK1), which is important for cell migration and is impaired in ATP7A deficient breast cancer cells.^55^ FAK1 activation and myocyte migration are important for myotube formation,^56^ so impaired FAK1 activation could contribute to the aberrant myotube morphology of ATP7A deficient myoblasts differentiated on Matrigel. Other extracellular Cu-dependent enzymes like SOD3 are expressed in muscle, so future studies are needed to determine whether these secreted cuproenzymes may also contribute to the function of myoblasts and other cell types within the regenerating skeletal muscle niche.

This study is consistent with an overall model where Cu being an important driver of mammalian cellular differentiation and post-transcriptional control of *Atp7a* RNA contributes to differentiation-dependent Cu redistribution from the cytoplasm to secreted cuproenzymes via ATP7A protein. Our results provide a model of post-transcriptional regulation of Cu distribution in the context of cellular differentiation and new avenues to identify novel therapeutic strategies to modulate Cu levels in a tissue-specific manner.

## Materials and methods

### Growth and differentiation of cell lines in culture

C2C12, N2A, and NIH 3T3 cell lines were purchased from American Type Culture Collection (ATCC). C2C12, N2A, and 3T3 cells were grown in DMEM in the presence of 10% fetal bovine serum (FBS), 50 µg/ml penicillin and streptomycin (pen/strep), and 2.5 µg/ml Plasmocin treatment (InVivogen) at 37°C in 5% CO_2_. To induce C2C12 myoblast differentiation, cells were grown to 80% confluence and then switched to DMEM with 2% horse serum and 50 µg/ml pen/strep for 3–5 d. For experiments with added histidine or BSA, C2C12 myoblasts were differentiated in the presence of 20 µM His or 2% BSA added to normal differentiation medium. For experiments with added Cu or Cu chelation, cells were grown in 20 µM Cu, BCS, or TEPA for 4 d or differentiated with variable BCS (25, 50, or 100 µM) or TTM (1, 2, or 5 µM) for approximately 4 d. Myocyte (MC) samples were collected when the majority of cells were elongated and just beginning to fuse. The early myotube (eMT) samples were collected when the majority of control cells appeared as small myotubes with few mononucleated cells remaining. Differentiation to mature myotubes (MT) was considered complete when robust myotube formation was detected in control samples. Unless otherwise noted, C2C12 differentiation was performed on normal tissue culture coated dishes without additional extracellular matrix. For extracellular matrix rescue experiments, Matrigel (Corning) was diluted 1:2 in DMEM and used to coat dishes for 1 h at 37°C prior to plating control or *Atp7a* knockdown cells for differentiation.

### Inductively coupled plasma–optical emissions spectroscopy (ICP–OES)

Total metals were quantified using ICP–OES as previously described.^26^ At various stages of differentiation (MB, MC, eMT, MT), cells were washed three times in PBS, harvested by scraping, and collected by centrifugation. Pellets were digested in trace metals grade nitric acid and diluted in ultrapure water. Cu levels were normalized to zinc or sulfur and calcium levels were used as a positive control for differentiation. Values for *n* = 4 proliferating cells were averaged and Cu or calcium levels were reported as fold change relative to the average of proliferating cells.

### Immunofluorescence staining

Cells were washed in PBS and fixed with 3.7% formaldehyde for 10 min at room temperature. Cells were blocked in 3% BSA with 0.3% Trition-X-100 for 1 h at room temperature and then incubated with anti-eMyHC antibody (Supplementary Table S3) diluted 1:10 in 0.5X blocking buffer overnight at 4°C. The following morning, cells were washed and incubated with FITC-conjugated anti-mouse antibody (Jackson ImmunoResearch #715-545-151) diluted 1:500 in 0.5X blocking buffer for 1 h at room temperature. Nuclei were visualized using 4′-6-diamidino-2-phenylindole (DAPI). Cells were imaged using an Olympus IX83 inverted fluorescence microscope using a U Plan fluorite 10X phase objective lens (NA 0.3 WD 10 mm). Images were captured using a DP74 Color CMOS Camera (cooled 20.8 MP pixel-shift, 60 FPS) using CellSens Dimension V2 software. Images were subjected to 2D-deconvolution and exported as red green blue (RGB) tif files.

### Immunoblotting

Specific proteins were quantified by immunoblot as previously described.^57^ Cells were washed twice in PBS and then incubated in radioimmunoprecipitation buffer [10 mM piperazine-*N*-*N*-bis(2-ethanesulfonic acid)] supplemented with protease inhibitor (Pierce) and phosphatase inhibitor (Sigma Aldrich). Samples were sonicated on ice three times, 10 s each at ∼40% output on an ultrasonic membrane disruptor (Fisher Model 100). After sonication, samples were incubated on ice for 30 min and centrifuged at 21 000× *g* for 30 min. The supernatant was transferred to new tubes and total protein concentration was determined using Bradford assay (Bio-Rad). To prepare for SDS-PAGE, protein was diluted in Laemmli sample buffer (Bio-Rad) and boiled for 5 min. For immunoblots probed for ATP7A and phosphorylated proteins, samples were not boiled. For immunoblots probed for phosphorylated proteins, samples were heated to 55°C for 5 min. Proteins were separated on TGX stain-free 4–20% polyacrylamide gels and then transferred to nitrocellulose membrane using a Trans-Blot Turbo semi-dry transfer system (Bio-Rad). Unless otherwise noted, gels were loaded with 20 µg of lysate. After transfer, blots were blocked in tris buffered saline with 0.1% Tween-20 (TBST) containing 5% milk or in Every Blot blocking reagent (Bio-Rad). Proteins of interest were then probed with primary antibodies (Supplementary Table S3) overnight at 4°C with gentle agitation and then probed with horseradish peroxidase (HRP)-conjugated secondary antibodies diluted 1:10 000 in TBST for 1 h at room temperature (Jackson ImmunoResearch, #111-035-003 for anti-rabbit, #115-035-003 for anti-mouse). Target proteins were visualized using Pico Plus ECL substrate (ThermoFisher) on a Bio-Rad Chemi-doc imager. Immunoblots were quantified by densitometry using ImageLab software (Bio-Rad) and normalized to total protein as quantified using stain-free gel imaging (Bio-Rad) or Ponceau stain.

### RNA isolation, reverse transcription, and quantitative PCR

Steady-state transcript levels were determined using quantitative PCR on reverse-transcribed cDNAs (RT-qPCR) as previously described.^57^ RNA was isolated using TriZol (Invitrogen) according to the manufacturer's instructions. cDNA was synthesized from 500 ng of RNA using the Maxima First Strand cDNA Synthesis Kit with dsDnase (ThermoFisher). Quantitative PCR was performed using SYBR Select Master Mix (Applied Biosystems) on a QuantStudio 3 Real Time PCR system (Applied Biosystems). Primer sequences can be found in Supplementary Table S4. Results were quantified using the comparative Ct method with *Rplp0* used as a normalizer.^58^

### RNA stability assays

RNA stability was analysed by actinomycin D chase assay as previously described.^26^ Briefly, cells were treated with 5 µg/µl actinomycin D to stop transcription and samples were harvested after 0.25, 2, 4, or 6 h. Samples were harvested in TriZol and analysed by qRT-PCR. *Rplp0* was used as a normalizer and *Myc* or *18* *s rRNA* was used as a positive control for actinomycin D. RNA half-life was calculated in GraphPad Prism using nonlinear requression and one-phase decay equation as previously described.^32^ Data points from *n* = 3 experiments were plotted and statistical analysis comparing myocytes, myotubes, and Cu- or TEPA-treated myoblasts to nontreated myoblasts was performed using the extra sum-of-squares *F*-test.

### Atp7a 3′ UTR annotation and luciferase reporter constructs

The *Atp7a* 3′ UTR was identified using the UCSC Genome Browser (RefSeq: NM_009726.5). Polyadenylation sites were annotated using PolyA_DB3^59^ and ARE were identified manually using the minimal ARE nonamer *WWAUUUAWW* where W is either A or U.^60^ Luciferase constructs were used as previously described.^32^ To make reporter constructs, the *Atp7a* 3′ UTR was synthesized with restriction endonuclease sites for molecular cloning (Genewiz/Azenta Life Science). To generate luciferase reporters, we used an approach similar to that used to generate *Pabpn1* 3′ UTR containing reporters.^32^ Briefly, we subcloned the firefly luciferase gene from the pGL3basic plasmid (Promega) downstream of the pCMV promoter in a pcDNA3.1 plasmid (AddGene) to generate pcLuci reporters. We then cloned the *Atp7a* 3′ UTR downstream of the firefly luciferase gene to generate pcLuci-*Atp7a* 3′ UTR. PCR was used to amplify the *Atp7a* 3′ UTR1 fragment which was subcloned downstream of firefly luciferase in pcLuci. All constructs were confirmed by sequencing. Control constructs containing the *Pabpn1* 3′ UTR (pcLuci-*Pabpn1* 3′ UTR) were generously provided by Dr Anita Corbett (Emory University).^32^

### Luciferase activity assays

Proliferating C2C12 myoblasts, N2A, and 3T3 cells were transfected with pcLuci (control), pcLuci-Atp7a 3′ UTR/UTR1, or pcLuci-Pabpn1 3′ UTR along with the pRL-CMV Renilla luciferase loading control vector (Promega) using lipofectamine 3000 (Invitrogen) according to the manufacturer's instructions. Cells were incubated for 24 h and then harvested for analysis using the Dual-Luciferase Reporter Assay System (Promega) according to the manufacturer's instructions. Luminescence was read on a BioTek Gen5 plate reader and all data are reported as a ratio of firefly/Renilla luciferase activity. For C2C12 myotubes, myoblasts were transfected and induced to differentiate, and then analysed as described earlier.

### Dicer-substrate siRNA knockdowns

The functional importance of ATP7A and ATOX1 was determined by knockdown using dicer substrate siRNAs (IDT). Cells were transfected with 100 nM siRNA or nontargeting negative control siRNA with Lipofectamine 3000 (Invitrogen) according to the manufacturer's protocol. Briefly, C2C12 cells were grown to 60% confluence and then incubated with transfection mixes containing 100 nM *Atp7a* or *Atox1* targeting siRNA, negative control siRNA (IDT NC-1), or lipofectamine/opti-mem only in DMEM with 10% FBS without antibiotics overnight at 37°C. After 16–18 h, transfection medium was replaced with normal growth medium or differentiation medium.

### Primary myoblast isolation, culture, and differentiation

All mouse procedures were performed with approval of the University of Missouri Animal Care and Use Committee. Primary muscle stem cell-derived myoblasts were isolated from hindlimb muscles of five combined male *Atp7a^fl/y^* mice using magnetic cell sorting as previously described.^57^ Muscle tissue was rinsed in PBS, minced, and then digested in DMEM with 0.1% Pronase (EMD Millipore) and 25 mM 4-(2-hydroxyethyl)-1-piperazineethanesulfonic acid (HEPES) pH 7.4 for 1 h at 37°C with gentle agitation. Digests were centrifuged and resuspended in DMEM containing 10% FBS and pen/strep, triturated using a 25 ml serological pipette and insoluble material was separated using 100 µM Steriflip filters (EMD Millipore). After filtering, the remaining muscle digest was resuspended in ammonium-chloride-potassium lysis buffer (Gibco) to remove residual red blood cells. Cells were washed with 0.5% BSA containing 2 mM EDTA and labeled with biotin-conjugated antibodies to CD31, CD45, and Sca1 (Satellite Cell isolation kit, Miltenyi Biotec). The CD31^+^/CD45^+^/Sca1^+^ cells were removed using magnetic streptavidin beads and the unbound myoblasts were collected and plated on dishes coated with Collagen I (Gibco) in normal growth medium [Ham's F10 (Cytiva) with 20% FBS, 50 µg/ml pen/strep, 2.5 µg/ml Plasmocin, and 5 ng/ml basic fibroblast growth factor (PeproTech)]. To induce Cre-mediated recombination, proliferating *Atp7a^fl/y^*cells were treated with adenoviral Cre (Ad-Cre-green fluorescent protein (GFP), Vector Biolabs Cat. #1700) at an MOI of 1 and incubated overnight in normal growth medium. Transduction was assessed by observing GFP signal using microscopy and efficiency of Cre-mediated recombination was determined by qRT-PCR and immunoblot. Differentiation was induced by plating primary myoblasts on dishes coated with enactin-collagen-laminin solution (Sigma) for 1 h. Cells were differentiated in DMEM plus 1% insulin-transferrin-selenium solution (Gibco) for 72 h.

### Cell proliferation assays using EdU

Proliferation of *Atp7a^fl/y^* and *Atp7a^fl/y^* + Cre cells was measured using ClickIt-EdU labeling (Invitrogen). Equal numbers of cells were plated on collagen-coated dishes and grown overnight. Cells were then treated with 10 µM EdU in normal growth medium and incubated at 37°C for 3 h. Cells were then washed, fixed, permeabilized and stained according to the manufacturer's instructions. Nuclei were labeled with DAPI and cells were imaged on an Olympus IX83 inverted fluorescence microscope. Total nuclei and EdU^+^ nuclei were counted using FIJI and proliferating cells were reported as percentage EdU^+^.

### LOX activity assay, inhibition, and rescue

Extracellular LOX activity was quantified using a fluorometric activity assay kit (Abcam Cat. #112139) as previously described.^55^ Prior to activity assay, cells were switched to DMEM with no phenol red supplemented with 4 mM L-Glutamine, 10% FBS, and 1x pen/strep for 24 h. To measure extracellular LOX activity, conditioned medium was harvested directly from the plate. Activity assays were performed using 50 µl of conditioned medium mixed with 50 µl of reaction mix in a black-walled 96 well plate. Reactions were incubated for 30 min at 37°C protected from light and then the fluorescent signal (Ex540nm/Em590nm) was quantified on a BioTek Gen5 plate reader (Ex540 nm/Em590 nm). In all cases, signal from a medium-only control well was subtracted as the blank and recombinant human LOXL2 (R&D Systems Cat. # 2639-AO-010) was used as a positive control. To inhibit LOX, βAPN resuspended in water was added to growth or differentiation medium at a final concentration of 1, 2, 5, or 10 mM for the duration of differentiation. LOX rescue experiments were performed using 1 µg/ml recombinant human LOX (Origene Technologies Cat. #TP313323) or recombinant human LOXL2 added directly to differentiation medium for 4 d.

### Statistical analysis

Statistical analysis was performed using GraphPad Prism 9.0. Data shown are reported as mean ± standard deviation for at least *n* = 3 experiments unless otherwise noted. For all figures, legends note statistical method employed, ranges for *P*-values, and sample size. For experiments employing one variable, statistical analysis was performed using Student's *t*-test. For experiments comparing two or more variables, one-way or two-way ANOVA tests were used as described in the figure legend. In all cases, *P* < 0.05 was considered to be statistically significant.

## Supplementary Material

mfad042_Supplemental_File

## Data Availability

The majority of data underlying this study are available in the manuscript and supplementary material. Additional raw data and replicate data are available will be shared on reasonable request to the corresponding author.

## References

[1] Tsang T. , Davis C. I., Brady D. C., Copper Biology, Curr. Biol., 2021, 31(9), R421–R427. 10.1016/j.cub.2021.03.054 33974864

[2] Kaler S. G. , Inborn Errors of Copper Metabolism, Handb. Clin. Neurol, 2013, 113, 1745–1754. 10.1016/B978-0-444-59565-2.00045-9 23622398 PMC4214864

[3] Ge E. J. , Bush A. I., Casini A., Cobine P. A., Cross J. R., DeNicola G. M., Dou Q. P., Franz K. J., Gohil V. M., Gupta S., Kaler S. G., Lutsenko S., Mittal V., Petris M. J., Polishchuk R., Ralle M., Schilsky M. L., Tonks N. K., Vahdat L. T., Van Aelst L., Xi D., Yuan P., Brady D. C., Chang C. J., Connecting Copper and Cancer: from Transition Metal Signalling to Metalloplasia, Nat. Rev. Cancer., 2022, 22(2), 102–113. 10.1038/s41568-021-00417-2 34764459 PMC8810673

[4] Turski M. L. , Brady D. C., Kim H. J., Kim B. E., Nose Y., Counter C. M., Winge D. R., Thiele D. J., A Novel Role for Copper in Ras/Mitogen-Activated Protein Kinase Signaling, Mol. Cell. Biol., 2012, 32(7), 1284–1295. 10.1128/MCB.05722-11 22290441 PMC3302449

[5] Tsang T. , Posimo J. M., Gudiel A. A., Cicchini M., Feldser D. M., Brady D. C., Copper Is an Essential Regulator of the Autophagic Kinases ULK1/2 to Drive Lung Adenocarcinoma, Nat. Cell. Biol, 2020, 22(4), 412–424. 10.1038/s41556-020-0481-4 32203415 PMC7610258

[6] Chojnowski J. E. , Li R., Tsang T., Alfaran F. H., Dick A., Cocklin S., Brady D. C., Strochlic T. I., Copper Modulates the Catalytic Activity of Protein Kinase CK2, Front. Mol. Biosci., 2022, 9, 878652. 10.3389/fmolb.2022.878652 35755824 PMC9224766

[7] Guo J. , Cheng J., Zheng N., Zhang X., Dai X., Zhang L., Hu C., Wu X., Jiang Q., Wu D., Okada H., Pandolfi P. P., Wei W., Copper Promotes Tumorigenesis by Activating the PDK1-AKT Oncogenic Pathway in a Copper Transporter 1 Dependent Manner, Adv. Sci., 2021, 8(18), 2004303. 10.1002/advs.202004303 PMC8456201 34278744

[8] Opazo C. M. , Lotan A., Xiao Z., Zhang B., Greenough M. A., Lim C. M., Trytell H., Ramírez A., Ukuwela A. A., Mawal C. H., McKenna J., Saunders D. N., Burke R., Gooley P. R., Bush A. I., Nutrient Copper Signaling Promotes Protein Turnover by Allosteric Activation of Ubiquitin E2D Conjugases, BioRXIV, 2021. 10.1101/2021.02.15.431211

[9] Krishnamoorthy L. , Cotruvo J. A. Jr., Chan J., Kaluarachchi H., Muchenditsi A., Pendyala V. S., Jia S., Aron A. T., Ackerman C. M., Wal M. N., Guan T., Smaga L. P., Farhi S. L., New E. J., Lutsenko S., Chang C. J., Copper Regulates Cyclic-AMP-Dependent Lipolysis, Nat. Chem. Biol., 2016, 12(8), 586–592. 10.1038/nchembio.2098 27272565 PMC4955676

[10] Foster A. W. , Young T. R., Chivers P. T., Robinson N. J., Protein Metalation in Biology, Curr. Opin. Chem. Biol., 2022, 66, 102095. 10.1016/j.cbpa.2021.102095 34763208 PMC8867077

[11] Bhattacharjee A. , Yang H., Duffy M., Robinson E., Conrad-Antoville A., Lu Y. W., Capps T., Braiterman L., Wolfgang M., Murphy M. P., Yi L., Kaler S. G., Lutsenko S., Ralle M., The Activity of Menkes Disease Protein ATP7A Is Essential for Redox Balance in Mitochondria, J. Biol. Chem., 2016, 291(32), 16644–16658. 10.1074/jbc.M116.727248 27226607 PMC4974379

[12] Tsvetkov P. , Coy S., Petrova B., Dreishpoon M., Verma A., Abdusamad M., Rossen J., Joesch-Cohen L., Humeidi R., Spangler R. D., Eaton J. K., Frenkel E., Kocak M., Corsello S. M., Lutsenko S., Kanarek N., Santagata S., Golub T. R., Copper Induces Cell Death by Targeting Lipoylated TCA Cycle Proteins, Science, 2022, 375(6586), 1254–1261. 10.1126/science.abf0529 35298263 PMC9273333

[13] Lutsenko S. , Dynamic and Cell-Specific Transport Networks for Intracellular Copper Ions, J. Cell. Sci., 2021, 134(21), 10.1242/jcs.240523 PMC8627558 34734631

[14] Dodani S. C. , Leary S. C., Cobine P. A., Winge D. R., Chang C. J., A Targetable Fluorescent Sensor Reveals that Copper-Deficient SCO1 and SCO2 Patient Cells Prioritize Mitochondrial Copper Homeostasis, J. Am. Chem. Soc., 2011, 133(22), 8606–8616. 10.1021/ja2004158 21563821 PMC3106114

[15] Cobine P. A. , Ojeda L. D., Rigby K. M., Winge D. R., Yeast Contain a Non-Proteinaceous Pool of Copper in the Mitochondrial Matrix, J. Biol. Chem., 2004, 279(14), 14447–14455. 10.1074/jbc.M312693200 14729672

[16] Culotta V. C. , Klomp L. W., Strain J., Casareno R. L., Krems B., Gitlin J. D., The Copper Chaperone for Superoxide Dismutase, J. Biol. Chem., 1997, 272(38), 23469–23472. 10.1074/jbc.272.38.23469 9295278

[17] Grasso M. , Bond G. J., Kim Y. J., Boyd S., Matson Dzebo M., Valenzuela S., Tsang T., Schibrowsky N. A., Alwan K. B., Blackburn N. J., Burslem G. M., Wittung-Stafshede P., Winkler D. D., Marmorstein R., Brady D. C., The Copper Chaperone CCS Facilitates Copper Binding to MEK1/2 to Promote Kinase Activation, J. Biol. Chem., 2021, 297(6), 101314. 10.1016/j.jbc.2021.101314 34715128 PMC8661025

[18] Hatori Y. , Lutsenko S., An Expanding Range Of Functions for the Copper Chaperone/Antioxidant Protein Atox1, Antioxid. Redox Signal., 2013, 19(9), 945–957. 10.1089/ars.2012.5086 23249252 PMC3763234

[19] Kim Y. J. , Bond G. J., Tsang T., Posimo J. M., Busino L., Brady D. C., Copper Chaperone ATOX1 Is Required for MAPK Signaling and Growth in BRAF Mutation-Positive Melanoma, Metallomics, 2019, 11(8), 1430–1440. 10.1039/c9mt00042a 31317143 PMC6693990

[20] Boulet A. , Vest K. E., Maynard M. K., Gammon M. G., Russell A. C., Mathews A. T., Cole S. E., Zhu X., Phillips C. B., Kwong J. Q., Dodani S. C., Leary S. C., Cobine P. A., The Mammalian Phosphate Carrier SLC25A3 Is a Mitochondrial Copper Transporter Required for Cytochrome c Oxidase Biogenesis, J. Biol. Chem., 2018, 293(6), 1887–1896. 10.1074/jbc.RA117.000265 29237729 PMC5808751

[21] Gudekar N. , Shanbhag V., Wang Y., Ralle M., Weisman G. A., Petris M. J., Metallothioneins Regulate ATP7A Trafficking and Control Cell Viability during Copper Deficiency and Excess, Sci. Rep., 2020, 10(1), 7856. 10.1038/s41598-020-64521-3 32398691 PMC7217913

[22] Petris M. J. , Mercer J. F., Culvenor J. G., Lockhart P., Gleeson P. A., Camakaris J., Ligand-Regulated Transport of the Menkes Copper P-Type ATPase Efflux Pump from the Golgi Apparatus to the Plasma Membrane: a Novel Mechanism of Regulated Trafficking, EMBO J., 1996, 15(22), 6084–6095. 10.1002/j.1460-2075.1996.tb00997.x 8947031 PMC452430

[23] Schaefer M. , Hopkins R. G., Failla M. L., Gitlin J. D., Hepatocyte-specific localization and copper-dependent trafficking of the Wilson's disease protein in the liver, Am. J. Physiol., 1999, 276(3), G639–G646. 10070040 10.1152/ajpgi.1999.276.3.G639

[24] Relaix F. , Bencze M., Borok M. J., Der Vartanian A., Gattazzo F., Mademtzoglou D., Perez-Diaz S., Prola A., Reyes-Fernandez P. C., Rotini A., Taglietti T., Perspectives on Skeletal Muscle Stem Cells, Nat. Commun., 2021, 12(1), 692. 10.1038/s41467-020-20760-6 33514709 PMC7846784

[25] Tavera-Montanez C. , Hainer S. J., Cangussu D., Gordon S. J. V., Xiao Y., Reyes-Gutierrez P., Imbalzano A. N., Navea J. G., Fazzio T. G., Padilla-Benavides T., The Classic Metal-Sensing Transcription Factor MTF1 Promotes Myogenesis in Response to Copper, FASEB J., 2019, 33(12), 14556–14574. 10.1096/fj.201901606R 31690123 PMC6894080

[26] Vest K. E. , Paskavitz A. L., Lee J. B., Padilla-Benavides T., Dynamic Changes in Copper Homeostasis and Post-Transcriptional Regulation of Atp7a during Myogenic Differentiation, Metallomics, 2018, 10(2), 309–322. 10.1039/C7MT00324B 29333545 PMC5824686

[27] Collins J. F. , Copper, in Present Knowledge in Nutrition, Marriott D.F.B. Bernadette P., Stallings Virginia A., Yates Allison A., eds. 2020, Cambridge, Massachusetts: Academic Press. p. 409–427.

[28] Gabay Yehezkely R. , Zaffryar-Eilot S., Kaganovsky A., Fainshtain Malka N., Aviram R., Livneh I., Hasson P., Intracellular Role for the Matrix-Modifying Enzyme Lox in Regulating Transcription Factor Subcellular Localization and Activity in Muscle Regeneration, Dev. Cell., 2020, 53(4), 406–417 e5. 10.1016/j.devcel.2020.04.002 32359406

[29] Kutchuk L. , Laitala A., Soueid-Bomgarten S., Shentzer P., Rosendahl A. H., Eilot S., Grossman M., Sagi I., Sormunen R., Myllyharju J., Maki J. M., Hasson P., Muscle Composition Is Regulated by a Lox-TGFbeta Feedback Loop, Development, 2015, 142(5), 983–993. 10.1242/dev.113449 25715398

[30] Bertinato J. , L'Abbe M. R., Copper Modulates the Degradation of Copper Chaperone for Cu, Zn Superoxide Dismutase by the 26 S Proteosome, J. Biol. Chem., 2003, 278(37), 35071–35078. 10.1074/jbc.M302242200 12832419

[31] Bertinato J. , Iskandar M., L'Abbe M. R., Copper Deficiency Induces the Upregulation of the Copper Chaperone for Cu/Zn Superoxide Dismutase in Weanling Male Rats, J. Nutr., 2003, 133(1), 28–31. 10.1093/jn/133.1.28 12514262

[32] Phillips B. L. , Banerjee A., Sanchez B. J., Di Marco S., Gallouzi I. E., Pavlath G. K., Corbett A. H., Post-Transcriptional Regulation of Pabpn1 by the RNA Binding Protein HuR, Nucleic Acids Res., 2018, 46(15), 7643–7661. 10.1093/nar/gky535 29939290 PMC6125628

[33] Wernimont A. K. , Huffman D. L., Lamb A. L., O'Halloran T. V., Rosenzweig A. C., Structural Basis for Copper Transfer by the Metallochaperone for the Menkes/Wilson Disease Proteins, Nat. Struct. Biol., 2000, 7(9), 766–771. 10966647 10.1038/78999

[34] Svetlana Lutsenko S. J. , Dmitriev O. Y., Chapter 5 - Molecular Architecture of the Copper-Transporting ATPase ATP7B, in Clinical and Translational Perspectives on Wilson Disease, Nanda Kerkar E.A.R., ed., 2019, Cambridge, Massachusetts: AcademicPress. p. 33–43.

[35] Grimsby J. L. , Lucero H. A., Trackman P. C., Ravid K., Kagan H. M., Role of Lysyl Oxidase Propeptide in Secretion and Enzyme Activity, J. Cell. Biochem., 2010, 111(5), 1231–1243. 10.1002/jcb.22845 20717923 PMC3858906

[36] Okada K. , Moon H. J., Finney J., Meier A., Mure M., Extracellular Processing of Lysyl Oxidase-like 2 and Its Effect on Amine Oxidase Activity, Biochemistry, 2018, 57(51), 6973–6983. 10.1021/acs.biochem.8b01008 30499665 PMC6548334

[37] Murtha K. , Hwang M., Peccarelli M. C., Scott T. D., Kebaara B. W., The Nonsense-Mediated mRNA Decay (NMD) Pathway Differentially Regulates COX17, COX19 and COX23 mRNAs, Curr. Genet., 2019, 65(2), 507–521. 10.1007/s00294-018-0892-y 30317392 PMC6420912

[38] Wang X. , Okonkwo O., Kebaara B. W., Physiological Basis of Copper Tolerance of *Saccharomyces Cerevisiae* Nonsense-Mediated mRNA Decay Mutants, Yeast, 2013, 30(5), 179–190. 10.1002/yea.2950 23450501

[39] Yu Z. , Cao W., Ren Y., Zhang Q., Liu J., ATPase Copper Transporter A, Negatively Regulated by miR-148a-3p, Contributes to Cisplatin Resistance in Breast Cancer Cells, Clin. Transl. Med., 2020, 10(1), 57–73. 10.1002/ctm2.19 32508020 PMC7240853

[40] Xiao F. , Xiao S., Xue M., miR-139 Controls Viability of Ovarian Cancer Cells Through Apoptosis Induction and Exosome Shedding Inhibition by Targeting ATP7A, OTT, 2019, 2019(12), 10727–10737. 10.2147/OTT.S221236 PMC6904246 31839712

[41] Song L. , Li Y., Li W., Wu S., Li Z., miR-495 Enhances the Sensitivity of Non-Small Cell Lung Cancer Cells to Platinum by Modulation of Copper-Transporting P-type Adenosine Triphosphatase A (ATP7A), J. Cell. Biochem., 2014, 115(7), 1234–1242. 10.1002/jcb.24665 24038379

[42] Cheng C. , Ding Q., Zhang Z., Wang S., Zhong B., Huang X., Shao Z., PTBP1 Modulates Osteosarcoma Chemoresistance to Cisplatin by Regulating the Expression of the Copper Transporter SLC31A1, J. Cell. Mol. Med., 2020, 24(9), 5274–5289. 10.1111/jcmm.15183 32207235 PMC7205786

[43] Malinouski M. , Hasan N. M., Zhang Y., Seravalli J., Lin J., Avanesov A., Lutsenko S., Gladyshev V. N., Genome-Wide RNAi Ionomics Screen Reveals New Genes and Regulation of Human Trace Element Metabolism, Nat. Commun., 2014, 5:1, 3301. 10.1038/ncomms4301 24522796 PMC5578452

[44] McCann C. J. , Hasan N. M., Padilla-Benavides T., Roy S., Lutsenko S., Heterogeneous Nuclear Ribonucleoprotein hnRNPA2/B1 Regulates the Abundance of the Copper-Transporter ATP7A in an Isoform-Dependent Manner, Front. Mol. Biosci., 2022, 9: p. 1067490. 10.3389/fmolb.2022.1067490 36545508 PMC9762481

[45] Wheeler J. R. , Whitney O. N., Vogler T. O., Nguyen E. D., Pawlikowski B., Lester E., Cutler A., Elston T., Dalla Betta N., Parker K. R., Yost K. E., Vogel H., Rando T. A., Chang H. Y., Johnson A. M., Parker R., Olwin B. B., RNA-Binding Proteins Direct Myogenic Cell Fate Decisions. Elife, 2022, 11: p. e75844. 10.7554/eLife.75844 PMC9191894 35695839

[46] Apponi L. H. , Corbett A. H., Pavlath G. K., RNA-Binding Proteins and Gene Regulation in Myogenesis, Trends Pharmacol. Sci., 2011, 32(11), 652–658. 10.1016/j.tips.2011.06.004 21982546 PMC3214663

[47] Shi D. L. , Grifone R., RNA-Binding Proteins in the Post-transcriptional Control of Skeletal Muscle Development, Regeneration and Disease, Front. Cell Dev. Biol., 2021, 9, 738978. 10.3389/fcell.2021.738978 34616743 PMC8488162

[48] Hinkle E. R. , Wiedner H. J., Black A. J., Giudice J., RNA Processing in Skeletal Muscle Biology and Disease, Transcription, 2019, 10(1), 1–20. 10.1080/21541264.2018.1558677 30556762 PMC6351125

[49] Kim H. J. , Mohassel P., Donkervoort S., Guo L., O'Donovan K., Coughlin M., Lornage X., Foulds N., Hammans S. R., Foley A. R., Fare C. M., Ford A. F., Ogasawara M., Sato A., Iida A., Munot P., Ambegaonkar G., Phadke R., O'Donovan D. G., Buchert R., Grimmel M., Topf A., Zaharieva I. T., Brady L., Hu Y., Lloyd T. E., Klein A., Steinlin M., Kuster A., Mercier S., Marcorelles P., Pereon Y., Fleurence E., Manzur A., Ennis S., Upstill-Goddard R., Bello L., Bertolin C., Pegoraro E., Salviati L., French C. E., Shatillo A., Raymond F. L., Haack T. B., Quijano-Roy S., Bohm J., Nelson I., Stojkovic T., Evangelista T., Straub V., Romero N. B., Laporte J., Muntoni F., Nishino I., Tarnopolsky M. A., Shorter J., Bonnemann C. G., Taylor J. P., Heterozygous Frameshift Variants in HNRNPA2B1 Cause Early-Onset Oculopharyngeal Muscular Dystrophy, Nat. Commun., 2022, 13(1), 2306. 10.1038/s41467-022-30015-1 35484142 PMC9050844

[50] Zhao W. , Zhang S., Zhu Y., Xi X., Bao P., Ma Z., Kapral T. H., Chen S., Zagrovic B., Yang Y. T., Lu Z. J., POSTAR3: an Updated Platform for Exploring Post-Transcriptional Regulation Coordinated by RNA-Binding Proteins, Nucleic Acids Res., 2022, 50(D1), D287–D294. 10.1093/nar/gkab702 34403477 PMC8728292

[51] Alipanahi B. , Delong A., Weirauch M. T., Frey B. J., Predicting the Sequence Specificities of DNA- and RNA-Binding Proteins by Deep Learning, Nat. Biotechnol, 2015, 33(8), 831–838. 10.1038/nbt.3300 26213851

[52] Yang H. , Ralle M., Wolfgang M. J., Dhawan N., Burkhead J. L., Rodriguez S., Kaplan J. H., Wong G. W., Haughey N., Lutsenko S., Copper-Dependent Amino Oxidase 3 Governs Selection of Metabolic Fuels in Adipocytes, PLoS Biol., 2018, 16(9), e2006519. 10.1371/journal.pbio.2006519 30199530 PMC6130853

[53] Hatori Y. , Yan Y., Schmidt K., Furukawa E., Hasan N. M., Yang N., Liu C. N., Sockanathan S., Lutsenko S., Neuronal Differentiation Is Associated With a Redox-Regulated Increase of Copper Flow to the Secretory Pathway, Nat. Commun., 2016, 7:1, 10640. 10.1038/ncomms10640 26879543 PMC4757759

[54] Wang S. X. , Mure M., Medzihradszky K. F., Burlingame A. L., Brown D. E., Dooley D. M., Smith A. J., Kagan H. M., Klinman J. P., A Crosslinked Cofactor in Lysyl Oxidase: Redox Function for Amino Acid Side Chains, Science, 1996, 273(5278), 1078–1084. 10.1126/science.273.5278.1078 8688089

[55] Shanbhag V. , Jasmer-McDonald K., Zhu S., Martin A. L., Gudekar N., Khan A., Ladomersky E., Singh K., Weisman G. A., Petris M. J., ATP7A Delivers Copper to the Lysyl Oxidase Family of Enzymes and Promotes Tumorigenesis And Metastasis, Proc. Natl. Acad. Sci. U.S.A. 2019, 116(14), 6836–6841. 10.1073/pnas.1817473116 30890638 PMC6452744

[56] Quach N. L. , Biressi S., Reichardt L. F., Keller C., Rando T. A., Focal Adhesion Kinase Signaling Regulates the Expression of Caveolin 3 And Beta1 Integrin, Genes Essential for Normal Myoblast Fusion, MBoC, 2009, 20(14), 3422–3435. 10.1091/mbc.e09-02-0175 19458188 PMC2710835

[57] Zhang Y. , Zeuthen C., Zhu C., Wu F., Mezzell A. T., Whitlow T. J., Choo H. J., Vest K. E., Pharyngeal Pathology in a Mouse Model of Oculopharyngeal Muscular Dystrophy Is Associated With Impaired Basal Autophagy in Myoblasts, Front. Cell Dev. Biol., 2022, 10, 986930. 10.3389/fcell.2022.986930 36313551 PMC9614327

[58] Livak K. J. , Schmittgen T. D., Analysis of Relative Gene Expression Data Using Real-Time Quantitative PCR and the 2(-Delta Delta C(T)) Method, Methods, 2001, 25(4), 402–408. 10.1006/meth.2001.1262 11846609

[59] Wang R. , Zheng D., Yehia G., Tian B., A Compendium of Conserved Cleavage And Polyadenylation Events in Mammalian Genes, Genome Res., 2018, 28(10), 1427–1441. 10.1101/gr.237826.118 30143597 PMC6169888

[60] Zubiaga A. M. , Belasco J. G., Greenberg M. E., The nonamer UUAUUUAUU Is the Key AU-Rich Sequence Motif that Mediates mRNA Degradation, Mol. Cell. Biol., 1995, 15(4), 2219–2230. 10.1128/MCB.15.4.2219 7891716 PMC230450

