# Supplementary material for: Regulation of Atp7a RNA contributes to differentiation-dependent Cu redistribution in skeletal muscle cells
Source: Metallomics. 2023 Jun 30;15(7):mfad042. doi: 10.1093/mtomcs/mfad042 (PMC10339032; doi:10.1093/mtomcs/mfad042)
Supplement: mfad042_Supplemental_File [file mfad042_supplemental_file.pdf]

Figure S1

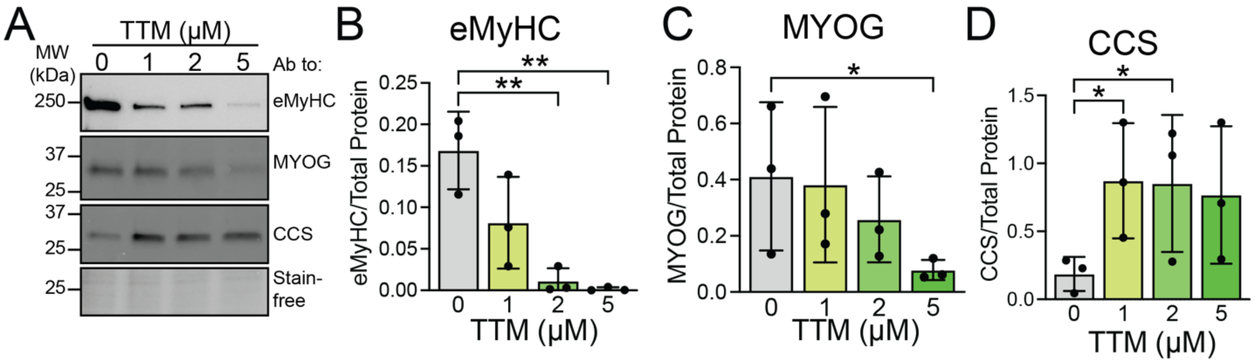

Supplementary Figure 1: TTM impairs C2C12 myoblast differentiation. ***A)*** Immunoblot of lysates from TTM-treated C2C12 myotubes probed with antibodies to eMyHC, myogenin (MYOG), and the Cu chaperone for SOD1 (CCS). Total protein is shown using Stain-free gel imaging technology. Quantification for eMyHC ***(B)***, MYOG ***(C)***, and CCS ***(D)*** as measured by densitometry and normalized to total protein showing significant decreases in MYOG and eMyHC and a significant increase in CCS with TTM treatment. Shown is the mean ± standard deviation for n = 3-4 experiments. For all experiments, statistical significance was determined using one-way ANOVA (* $p < 0.05$, ** $p < 0.01$, *** $p < 0.001$).

Figure S2

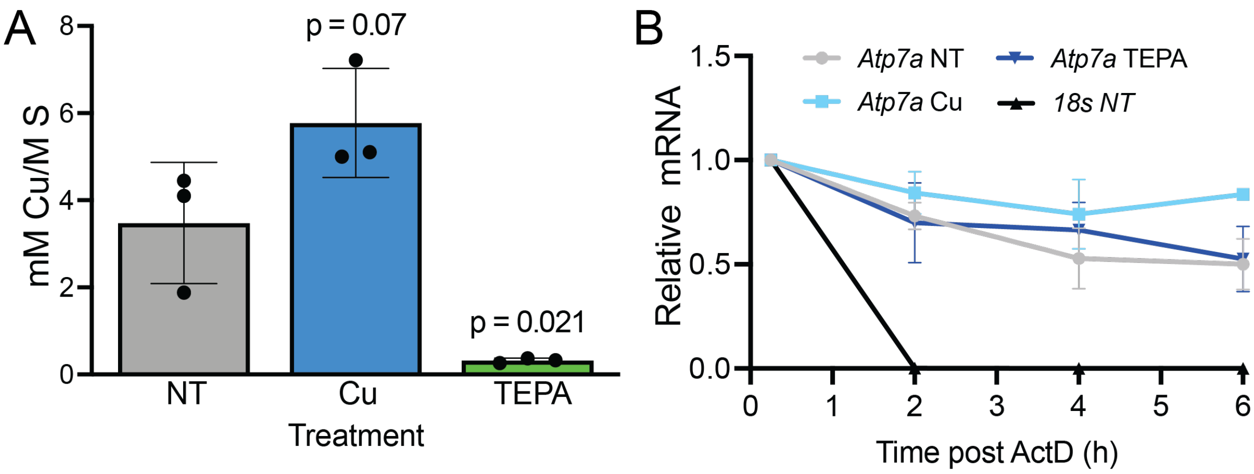

Supplementary Figure 2: Extended growth in TEPA decreases intracellular Cu and does not affect *Atp7a* RNA stability. ***A)*** Levels of Cu (Cu) normalized to sulfur (S) from C2C12 in myoblasts (MB) in normal growth medium (NT) or grown in medium containing 20 µM $CuSO_4$ (Cu) or 20 µM TEPA (TEPA) for four days. Values were measured using ICP-OES. ***B)*** Actinomycin D assay showing that *Atp7a* mRNA is stable in C2C12 myoblasts are grown in the presence of Cu but unstable in normal growth medium or TEPA-treated medium. Shown is mean ± standard deviation for n = 3 experiments. Statistical significance was determined by one-way ANOVA.

Figure S3

```
Reference sequence (1): mmAtp7a3pUTR
Identities normalised by aligned length.
Colored by: identity

                       cov    pid     1 [         .         .         .         .         :
1 mmAtp7a3pUTR      100.0% 100.0%       AAAGCCTTGGGGGATGCTGCTAGTTAAAGTTCACGTGCACTGACACAGCACTTAC
2 hsATP7A3pUTR       84.7%  47.6%       AAGGCCATGGAGAGTGCTGCCAGTTTAACTTGTCATGCACTGACACAGCATTCAT
  consensus/100%                        AAuGCCsTGGuGuuTGCTGCsAGTTsAAsTTssCuTGCACTGACACAGCAsTsAs
  consensus/90%                         AAuGCCsTGGuGuuTGCTGCsAGTTsAAsTTssCuTGCACTGACACAGCAsTsAs
  consensus/80%                         AAuGCCsTGGuGuuTGCTGCsAGTTsAAsTTssCuTGCACTGACACAGCAsTsAs
  consensus/70%                         AAuGCCsTGGuGuuTGCTGCsAGTTsAAsTTssCuTGCACTGACACAGCAsTsAs

                       cov    pid    81           .         1         .         .         .
1 mmAtp7a3pUTR      100.0% 100.0%       ---ATCTTATGTCAATCTCATGCTCTTACGTGCAGGATTCTATCTGAGTTGTATT
2 hsATP7A3pUTR       84.7%  47.6%       TTGTAGAAGGATTTTTCTCATGCTCTTATATTAGGGATTCTATTTGAGTTGCGTT
  consensus/100%                        ...sssssusuTsssTCTCATGCTCTTAsuTssuGGATTCTATsTGAGTTGsuTT
  consensus/90%                         ...sssssusuTsssTCTCATGCTCTTAsuTssuGGATTCTATsTGAGTTGsuTT
  consensus/80%                         ...sssssusuTsssTCTCATGCTCTTAsuTssuGGATTCTATsTGAGTTGsuTT
  consensus/70%                         ...sssssusuTsssTCTCATGCTCTTAsuTssuGGATTCTATsTGAGTTGsuTT

                       cov    pid   161           .         .         .         2         .
1 mmAtp7a3pUTR      100.0% 100.0%       CAGGGCAGGAGCTCTGAACCTGGCATTATCTAGAAGAAGTTCCCAGCAGTTTTTC
2 hsATP7A3pUTR       84.7%  47.6%       CAAGGCATCAGCTCTGAACCTAGCTTTATTTAAACTGAATTTCCAGTATATTTTT
  consensus/100%                        CAuGGCAssAGCTCTGAACCTuGCsTTATsTAuAssuAuTTsCCAGsAssTTTTs
  consensus/90%                         CAuGGCAssAGCTCTGAACCTuGCsTTATsTAuAssuAuTTsCCAGsAssTTTTs
  consensus/80%                         CAuGGCAssAGCTCTGAACCTuGCsTTATsTAuAssuAuTTsCCAGsAssTTTTs
  consensus/70%                         CAuGGCAssAGCTCTGAACCTuGCsTTATsTAuAssuAuTTsCCAGsAssTTTTs

                       cov    pid   241           :         .         .         .         .
1 mmAtp7a3pUTR      100.0% 100.0%       GAAC-AGTAAGTTTAGAACAAGCTCTATAGTTAGAAGTTGCTAATGTGCTGCTAA
2 hsATP7A3pUTR       84.7%  47.6%       GAGCAGTGAGGTTTACAACAAGCCCTACAATTAGAGATTGCTGAACTGCTGCTAA
  consensus/100%                        GAuC.ussAuGTTTAsAACAAGCsCTAsAuTTAGAuuTTGCTuAssTGCTGCTAA
  consensus/90%                         GAuC.ussAuGTTTAsAACAAGCsCTAsAuTTAGAuuTTGCTuAssTGCTGCTAA
  consensus/80%                         GAuC.ussAuGTTTAsAACAAGCsCTAsAuTTAGAuuTTGCTuAssTGCTGCTAA
  consensus/70%                         GAuC.ussAuGTTTAsAACAAGCsCTAsAuTTAGAuuTTGCTuAssTGCTGCTAA

                       cov    pid   321           .         .         :         .         .
1 mmAtp7a3pUTR      100.0% 100.0%       AAAAAAAAAAAAAAAAGTGCCCAAAGACAGGACGATTAAAAATGTGAA--------
2 hsATP7A3pUTR       84.7%  47.6%       AAA-----AAAAAAAAGGCCCAAGAAGAAGAAAATGAAAAATTTGAAGATTTGAC
  consensus/100%                        AAA.....AAAAAAAusGCCCAAuuAsAuGAsuATsAAAAATsTGAA........
  consensus/90%                         AAA.....AAAAAAAusGCCCAAuuAsAuGAsuATsAAAAATsTGAA........
  consensus/80%                         AAA.....AAAAAAAusGCCCAAuuAsAuGAsuATsAAAAATsTGAA........
  consensus/70%                         AAA.....AAAAAAAusGCCCAAuuAsAuGAsuATsAAAAATsTGAA........

                       cov    pid   401           .         .         .         .         :
1 mmAtp7a3pUTR      100.0% 100.0%       GAGCATGATTTTGAAGGTATTCTTGAGCCTATATACTTGCCCCACTGGGGAGCAA
2 hsATP7A3pUTR       84.7%  47.6%       ACTCAAAATATTGAAGATACTCTCAAGCCTGTATCCCTGCCCCACTGGGGAGCAA
  consensus/100%                        ussCAsuATsTTGAAGuTAsTCTsuAGCCTuTATsCsTGCCCCACTGGGGAGCAA
  consensus/90%                         ussCAsuATsTTGAAGuTAsTCTsuAGCCTuTATsCsTGCCCCACTGGGGAGCAA
  consensus/80%                         ussCAsuATsTTGAAGuTAsTCTsuAGCCTuTATsCsTGCCCCACTGGGGAGCAA
  consensus/70%                         ussCAsuATsTTGAAGuTAsTCTsuAGCCTuTATsCsTGCCCCACTGGGGAGCAA

                       cov    pid   481           .         5         .         .         .
1 mmAtp7a3pUTR      100.0% 100.0%       AC---CTAGTTTAGAAGGAAAACAGTTGCAACTGTCTCAAAACAGATGTGCCTTA
2 hsATP7A3pUTR       84.7%  47.6%       ACATCTAGTTTTAGAAGGGAAACAGTTGAAACTGTTTAAAAATAGATGTGCCTTA
  consensus/100%                        AC...ssusTTTAGAAGGuAAACAGTTGsAACTGTsTsAAAAsAGATGTGCCTTA
  consensus/90%                         AC...ssusTTTAGAAGGuAAACAGTTGsAACTGTsTsAAAAsAGATGTGCCTTA
```

```
   consensus/80%                      AC...ssusTTTAGAAGGuAAACAGTTGsAACTGTsTsAAAAsAGATGTGCCTTA
   consensus/70%                      AC...ssusTTTAGAAGGuAAACAGTTGsAACTGTsTsAAAAsAGATGTGCCTTA

                     cov     pid  561          .         .         .         6         .
 1 mmAtp7a3pUTR   100.0% 100.0%       ATAATTCATGTGTCATTGTCCTTTAGT-------TCCTAAGAGCCTGCAGGATGC
 2 hsATP7A3pUTR    84.7%  47.6%       TTCTCCCTGCATCCTTGTCCTTGCAGGTGCTTTTTTAGATGCTCCAATATGTCTT
   consensus/100%                     sTssssCssssssCsTsssCsTssAGs.......TsssAsGssCCsusAsGssss
   consensus/90%                      sTssssCssssssCsTsssCsTssAGs.......TsssAsGssCCsusAsGssss
   consensus/80%                      sTssssCssssssCsTsssCsTssAGs.......TsssAsGssCCsusAsGssss
   consensus/70%                      sTssssCssssssCsTsssCsTssAGs.......TsssAsGssCCsusAsGssss

                     cov     pid  641          :         .         .         .         .
 1 mmAtp7a3pUTR   100.0% 100.0%       CCAAGTTTAGGTGGTTTTTCCTTGATAAAAATAACTGACAACAATCACTTGGAAT
 2 hsATP7A3pUTR    84.7%  47.6%       CCAAGTTTAGGTGGTTTTTCATTGATTAAAAATAACTGACAACTGTTCTAATATT
   consensus/100%                     CCAAGTTTAGGTGGTTTTTCsTTGATsAAAAssAsssussAssssssCTsusAsT
   consensus/90%                      CCAAGTTTAGGTGGTTTTTCsTTGATsAAAAssAsssussAssssssCTsusAsT
   consensus/80%                      CCAAGTTTAGGTGGTTTTTCsTTGATsAAAAssAsssussAssssssCTsusAsT
   consensus/70%                      CCAAGTTTAGGTGGTTTTTCsTTGATsAAAAssAsssussAssssssCTsusAsT

                     cov     pid  721          .         .         :         .         .
 1 mmAtp7a3pUTR   100.0% 100.0%       GCCA----GACACTAAGACCCTGTCTTAAA-ACATTTTCGTAGCTGGCAGCTCTT
 2 hsATP7A3pUTR    84.7%  47.6%       GCTCAAAAGACCTTAAAGGTCTGTAGGGTTCCCTGCCTCCCATCTTTCCACTGTT
   consensus/100%                     GCss....GACssTAAuussCTGTsssuss.sCssssTCssAsCTssCsuCTsTT
   consensus/90%                      GCss....GACssTAAuussCTGTsssuss.sCssssTCssAsCTssCsuCTsTT
   consensus/80%                      GCss....GACssTAAuussCTGTsssuss.sCssssTCssAsCTssCsuCTsTT
   consensus/70%                      GCss....GACssTAAuussCTGTsssuss.sCssssTCssAsCTssCsuCTsTT

                     cov     pid  801          .         .         .         .         :
 1 mmAtp7a3pUTR   100.0% 100.0%       CTGTGTCGTTCAGAAGACCATAAGATCTACAGGGTTCTGTACCCTCTTGTTTCTC
 2 hsATP7A3pUTR    84.7%  47.6%       -------------------------------------------------------
   consensus/100%                     .......................................................
   consensus/90%                      .......................................................
   consensus/80%                      .......................................................
   consensus/70%                      .......................................................

                     cov     pid  881          .         9         .         .         .
 1 mmAtp7a3pUTR   100.0% 100.0%       TTCCTTCAGTTTCCCCAGCCCTGTGCTAGCTTTCAGTTCAGCTTTGTTCACAGTA
 2 hsATP7A3pUTR    84.7%  47.6%       TTCCTTCAAGTTTCCTAGCTCTGTGCTCAGTTTCAGTTCACTCCTGCCAAGTTGC
   consensus/100%                     TTCCTTCAusTTsCCsAGCsCTGTGCTsusTTTCAGTTCAssssTGsssAssssu
   consensus/90%                      TTCCTTCAusTTsCCsAGCsCTGTGCTsusTTTCAGTTCAssssTGsssAssssu
   consensus/80%                      TTCCTTCAusTTsCCsAGCsCTGTGCTsusTTTCAGTTCAssssTGsssAssssu
   consensus/70%                      TTCCTTCAusTTsCCsAGCsCTGTGCTsusTTTCAGTTCAssssTGsssAssssu

                     cov     pid  961          .         .         .         0         .
 1 mmAtp7a3pUTR   100.0% 100.0%       TCTCCAT--CAGTCC-CAGTCGAGAAACTTAGAACCTCTCTCACTGAAAAATTCT
 2 hsATP7A3pUTR    84.7%  47.6%       TGCTGATCTCAGTCTGGAAACTTAACATTATGAGCCTTTTCTGCTCAAAAAATTT
   consensus/100%                     TssssAT..CAGTCs.sAusCssuAsAsTssGAuCCTsTsssuCTsAAAAAsTsT
   consensus/90%                      TssssAT..CAGTCs.sAusCssuAsAsTssGAuCCTsTsssuCTsAAAAAsTsT
   consensus/80%                      TssssAT..CAGTCs.sAusCssuAsAsTssGAuCCTsTsssuCTsAAAAAsTsT
   consensus/70%                      TssssAT..CAGTCs.sAusCssuAsAsTssGAuCCTsTsssuCTsAAAAAsTsT

                     cov     pid 1041          :         .         .         .         .
 1 mmAtp7a3pUTR   100.0% 100.0%       CATATACGGGCCATAGAAAAGTATGTGGTCTCATTAAATTCATCTTTTGGGATAT
 2 hsATP7A3pUTR    84.7%  47.6%       CATATACAGGTCATATAAAATTACCTGGATTCACTAAATTTGTTTGTTGTTGTTC
   consensus/100%                     CATATACuGGsCATAsAAAAsTAssTGGssTCAsTAAATTsuTsTsTTGssuTss
   consensus/90%                      CATATACuGGsCATAsAAAAsTAssTGGssTCAsTAAATTsuTsTsTTGssuTss
   consensus/80%                      CATATACuGGsCATAsAAAAsTAssTGGssTCAsTAAATTsuTsTsTTGssuTss
   consensus/70%                      CATATACuGGsCATAsAAAAsTAssTGGssTCAsTAAATTsuTsTsTTGssuTss
```

```
                       cov     pid 1121         .         .         :         .         .
1 mmAtp7a3pUTR      100.0% 100.0%      --TCACTTGGCTGGTATATTATAATTTAATAAAATTTTAGTCGCCTCTGTTTTA
2 hsATP7A3pUTR       84.7%  47.6%      AGAGTCTTGTTTTGCAGCCCAGGCTGGAGTGCAGTGGCACCATCTTGGCTCACT
  consensus/100%                       ..sssCTTGssTsGsAssssAsusTssAuTusAuTsssAssssCsTsssTssss
  consensus/90%                        ..sssCTTGssTsGsAssssAsusTssAuTusAuTsssAssssCsTsssTssss
  consensus/80%                        ..sssCTTGssTsGsAssssAsusTssAuTusAuTsssAssssCsTsssTssss
  consensus/70%                        ..sssCTTGssTsGsAssssAsusTssAuTusAuTsssAssssCsTsssTssss

                       cov     pid 1201         .         .         .         .         :
1 mmAtp7a3pUTR      100.0% 100.0%      TATTCTCAT-------------------------------------------CG
2 hsATP7A3pUTR       84.7%  47.6%      AATTCTCCCTGCCTCAGCCTCCTGAGTAGCTAGGATTACAGGTGCCTGCCACCA
  consensus/100%                       sATTCTCss...........................................Cu
  consensus/90%                        sATTCTCss...........................................Cu
  consensus/80%                        sATTCTCss...........................................Cu
  consensus/70%                        sATTCTCss...........................................Cu

                       cov     pid 1281         .         3         .         .         .
1 mmAtp7a3pUTR      100.0% 100.0%      --------------------------------------TACACACCAAACTGTA
2 hsATP7A3pUTR       84.7%  47.6%      GTAGAGACGGGGTTTCGCCATGTTGGCTAGCCTGGTCTTAAACTCCTAACCTCA
  consensus/100%                       ......................................TAsACsCCsAACsssA
  consensus/90%                        ......................................TAsACsCCsAACsssA
  consensus/80%                        ......................................TAsACsCCsAACsssA
  consensus/70%                        ......................................TAsACsCCsAACsssA

                       cov     pid 1361         .         .         .         4         .
1 mmAtp7a3pUTR      100.0% 100.0%      AAATCAGAATATTCCTGGTTTATGATATCTT---GTAGTTAAGTATGTTGTTCT
2 hsATP7A3pUTR       84.7%  47.6%      AAGTGCTGGGATTACAGGTGTGAGCCTCCATGCCCAGCCTAAATTTGTATTTTT
  consensus/100%                       AAuTsssuusATTsCsGGTsTusGssssCsT...ssussTAAuTsTGTssTTsT
  consensus/90%                        AAuTsssuusATTsCsGGTsTusGssssCsT...ssussTAAuTsTGTssTTsT
  consensus/80%                        AAuTsssuusATTsCsGGTsTusGssssCsT...ssussTAAuTsTGTssTTsT
  consensus/70%                        AAuTsssuusATTsCsGGTsTusGssssCsT...ssussTAAuTsTGTssTTsT

                       cov     pid 1441         :         .         .         .         .
1 mmAtp7a3pUTR      100.0% 100.0%      TGTTTA------------------------------------------------
2 hsATP7A3pUTR       84.7%  47.6%      TATATATAATTTAATAGATTTTATTTATCTTTTAGTGTTTCAGATAACCTCTCA
  consensus/100%                       TuTsTA................................................
  consensus/90%                        TuTsTA................................................
  consensus/80%                        TuTsTA................................................
  consensus/70%                        TuTsTA................................................

                       cov     pid 1521         .         .         :         .         .
1 mmAtp7a3pUTR      100.0% 100.0%      ------------------------------------------------------
2 hsATP7A3pUTR       84.7%  47.6%      ACAAAGCTGCATTTACCAAAAAATACAGTAAAATCATAATACAGAAACTAAAAT
  consensus/100%                       ......................................................
  consensus/90%                        ......................................................
  consensus/80%                        ......................................................
  consensus/70%                        ......................................................

                       cov     pid 1601         .         .         .         .         :
1 mmAtp7a3pUTR      100.0% 100.0%      ------------------------------------------------------
2 hsATP7A3pUTR       84.7%  47.6%      TAAATATATACTCTTCTCTAGTTTAAAACATTTGAACTTGCCTAGTTAGTGTGG
  consensus/100%                       ......................................................
  consensus/90%                        ......................................................
  consensus/80%                        ......................................................
  consensus/70%                        ......................................................

                       cov     pid 1681         .         7         .         .         .
1 mmAtp7a3pUTR      100.0% 100.0%      ------------------------------------------------------
2 hsATP7A3pUTR       84.7%  47.6%      TTGCCAAATGGATTTAGAAATTCCCTTGTGAGTGCCTGGTAGCTAATACACTGG
```

```
   consensus/100%                    .......................................................
   consensus/90%                     .......................................................
   consensus/80%                     .......................................................
   consensus/70%                     .......................................................

                       cov    pid 1761        .         .         .         8         .
 1 mmAtp7a3pUTR    100.0% 100.0%     -------------------------------------------------------
 2 hsATP7A3pUTR     84.7%  47.6%     TTTAAATTTCTTTGTTAGTTGCAAGATGGATTTCATATGCAGAATATGTAAATGA
   consensus/100%                    .......................................................
   consensus/90%                     .......................................................
   consensus/80%                     .......................................................
   consensus/70%                     .......................................................

                       cov    pid 1841        :         .         .         .         .
 1 mmAtp7a3pUTR    100.0% 100.0%     TCTTTTATTTTCATGTACTT-------TAAGCTACTA--GCATAATGTCTAACAT
 2 hsATP7A3pUTR     84.7%  47.6%     CATTTTGTTCCCATTACCAGAAGCAAAGCTGCTGCTAACCCAACATCTGGCACAT
   consensus/100%                    ssTTTTuTTssCATsssCss.......sssGCTuCTA..sCAssATsTsssACAT
   consensus/90%                     ssTTTTuTTssCATsssCss.......sssGCTuCTA..sCAssATsTsssACAT
   consensus/80%                     ssTTTTuTTssCATsssCss.......sssGCTuCTA..sCAssATsTsssACAT
   consensus/70%                     ssTTTTuTTssCATsssCss.......sssGCTuCTA..sCAssATsTsssACAT

                       cov    pid 1921        .         .         :         .         .
 1 mmAtp7a3pUTR    100.0% 100.0%     --CATTTCTCCTTTTCAAGTCAATATTGAGTATAAAAATCATGTTTTATATTAC-
 2 hsATP7A3pUTR     84.7%  47.6%     TTCTTTTCTCCCCTTGAGGTCAGTAATAAATACAAAAAAATCATTTTTCTAGAGC
   consensus/100%                    ..CsTTTCTCCssTTsAuGTCAuTAsTuAuTAsAAAAAssssuTTTTsssssAs.
   consensus/90%                     ..CsTTTCTCCssTTsAuGTCAuTAsTuAuTAsAAAAAssssuTTTTsssssAs.
   consensus/80%                     ..CsTTTCTCCssTTsAuGTCAuTAsTuAuTAsAAAAAssssuTTTTsssssAs.
   consensus/70%                     ..CsTTTCTCCssTTsAuGTCAuTAsTuAuTAsAAAAAssssuTTTTsssssAs.

                       cov    pid 2001        .         .         .         .         :
 1 mmAtp7a3pUTR    100.0% 100.0%     GGAGAATGGGTTTTGTCCACTCCACCACTTTTCTCTCTTATTCCTTCACACCCAA
 2 hsATP7A3pUTR     84.7%  47.6%     GGGGATGGAGTTCTTCCTTTCCTACCCCTTTTCTCTTTTATCCTTTCATATATAC
   consensus/100%                    GGuGAssGuGTTsTssCssssCsACCsCTTTTCTCTsTTATsCsTTCAsAsssAs
   consensus/90%                     GGuGAssGuGTTsTssCssssCsACCsCTTTTCTCTsTTATsCsTTCAsAsssAs
   consensus/80%                     GGuGAssGuGTTsTssCssssCsACCsCTTTTCTCTsTTATsCsTTCAsAsssAs
   consensus/70%                     GGuGAssGuGTTsTssCssssCsACCsCTTTTCTCTsTTATsCsTTCAsAsssAs

                       cov    pid 2081        .         1         .         .         .
 1 mmAtp7a3pUTR    100.0% 100.0%     AATGCTGTCTTTCCAGATTTGGAAAAGACCTAACTTCTATCTTAGCTAT----CT
 2 hsATP7A3pUTR     84.7%  47.6%     CATGCTGT-CTTTCAGATTTAGAAAAGATCTAATTTCTGTCTCAGCTGTCTTAAA
   consensus/100%                    sATGCTGT.sTTsCAGATTTuGAAAAGAsCTAAsTTCTuTCTsAGCTuT....ss
   consensus/90%                     sATGCTGT.sTTsCAGATTTuGAAAAGAsCTAAsTTCTuTCTsAGCTuT....ss
   consensus/80%                     sATGCTGT.sTTsCAGATTTuGAAAAGAsCTAAsTTCTuTCTsAGCTuT....ss
   consensus/70%                     sATGCTGT.sTTsCAGATTTuGAAAAGAsCTAAsTTCTuTCTsAGCTuT....ss

                       cov    pid 2161        .         .         .         2         .
 1 mmAtp7a3pUTR    100.0% 100.0%     GTGCTATTAATCTAGAAAGGCAAACCTGTTTTAATGAAGTATGAATAAGTTTATA
 2 hsATP7A3pUTR     84.7%  47.6%     GTGCTATTAATCTAGAAAGGCAAACCCATTTCACTGAAATATCAATGGGTTTGCA
   consensus/100%                    GTGCTATTAATCTAGAAAGGCAAACCsuTTTsAsTGAAuTATsAATuuGTTTusA
   consensus/90%                     GTGCTATTAATCTAGAAAGGCAAACCsuTTTsAsTGAAuTATsAATuuGTTTusA
   consensus/80%                     GTGCTATTAATCTAGAAAGGCAAACCsuTTTsAsTGAAuTATsAATuuGTTTusA
   consensus/70%                     GTGCTATTAATCTAGAAAGGCAAACCsuTTTsAsTGAAuTATsAATuuGTTTusA

                       cov    pid 2241        :         .         .         .         .
 1 mmAtp7a3pUTR    100.0% 100.0%     TACTTATGCCGTCTTAAGAGTT-------TGAGTTTGTATATTTGTTTC-TTAAT
 2 hsATP7A3pUTR     84.7%  47.6%     TGCCTATGCCATCCTCCATGCTTTCAGTTTGAGTTTTATTATTTATTATTTTAAT
   consensus/100%                    TuCsTATGCCuTCsTssusGsT.......TGAGTTTsssTATTTuTTss.TTAAT
   consensus/90%                     TuCsTATGCCuTCsTssusGsT.......TGAGTTTsssTATTTuTTss.TTAAT
   consensus/80%                     TuCsTATGCCuTCsTssusGsT.......TGAGTTTsssTATTTuTTss.TTAAT
```

```
  consensus/70%                   TuCsTATGCCuTCsTssusGsT.......TGAGTTTsssTATTTuTTss.TTAAT

                   cov    pid 2321        .         .         :         .         .
1 mmAtp7a3pUTR   100.0% 100.0%    CA-TTGGCTTCTAGAAGAGAGAGCTAAACTAT-AGATGTCCAGTAGTT----TTT
2 hsATP7A3pUTR    84.7%  47.6%    CTTGTTTCTTCTAGAAGACAGAGCTGATAGGGTAAATGTTGAAAAAAGAAGCATC
  consensus/100%                  Cs.sTssCTTCTAGAAGAsAGAGCTuAsssus.AuATGTssAusAuss....sTs
  consensus/90%                   Cs.sTssCTTCTAGAAGAsAGAGCTuAsssus.AuATGTssAusAuss....sTs
  consensus/80%                   Cs.sTssCTTCTAGAAGAsAGAGCTuAsssus.AuATGTssAusAuss....sTs
  consensus/70%                   Cs.sTssCTTCTAGAAGAsAGAGCTuAsssus.AuATGTssAusAuss....sTs

                   cov    pid 2401        .         .         .         .         :
1 mmAtp7a3pUTR   100.0% 100.0%    AAGTCCCCCAGTCGTCAGCCAGTTTCTCCTTCTGTTCTGTATATGTTGTTGAGTC
2 hsATP7A3pUTR    84.7%  47.6%    AAGCA--GTTGAACACAGCCAGTTATTCTTCCATTATTATGTGTACCTTGGAGTC
  consensus/100%                  AAGss..sssGssssCAGCCAGTTssTCsTsCssTssTuTuTuTusssTsGAGTC
  consensus/90%                   AAGss..sssGssssCAGCCAGTTssTCsTsCssTssTuTuTuTusssTsGAGTC
  consensus/80%                   AAGss..sssGssssCAGCCAGTTssTCsTsCssTssTuTuTuTusssTsGAGTC
  consensus/70%                   AAGss..sssGssssCAGCCAGTTssTCsTsCssTssTuTuTuTusssTsGAGTC

                   cov    pid 2481        .         5         .         .         .
1 mmAtp7a3pUTR   100.0% 100.0%    ATAGGGCAGT------------------------------GAGAACAGCATTTC
2 hsATP7A3pUTR    84.7%  47.6%    GTGGGACAGTGGGAATAGCAGCTTGTAGTATTGAAATAATCAAAGAGATAATTTC
  consensus/100%                  uTuGGuCAGT..............................uAuuAsAssATTTC
  consensus/90%                   uTuGGuCAGT..............................uAuuAsAssATTTC
  consensus/80%                   uTuGGuCAGT..............................uAuuAsAssATTTC
  consensus/70%                   uTuGGuCAGT..............................uAuuAsAssATTTC

                   cov    pid 2561        .         .         .         6         .
1 mmAtp7a3pUTR   100.0% 100.0%    GTTG--GAGA---------------------------------------------
2 hsATP7A3pUTR    84.7%  47.6%    ATGCTAATTAGAAAAAGTCCTGCTTTAAGTAATATGTAGCCACATTTGAATCCTC
  consensus/100%                  uTss..ussA.............................................
  consensus/90%                   uTss..ussA.............................................
  consensus/80%                   uTss..ussA.............................................
  consensus/70%                   uTss..ussA.............................................

                   cov    pid 2641        :         .         .         .         .
1 mmAtp7a3pUTR   100.0% 100.0%    ----------------------GAGGCTGTATTCCTTAGAGAGGAGGGTCTTTTA
2 hsATP7A3pUTR    84.7%  47.6%    GAACTTTCATTAGCTTCAAAGAGAAGCTGTATTTACAGGAGAAAGAGG-TGATTA
  consensus/100%                  ......................GAuGCTGTATTssssuGAGAuuuuGG.sssTTA
  consensus/90%                   ......................GAuGCTGTATTssssuGAGAuuuuGG.sssTTA
  consensus/80%                   ......................GAuGCTGTATTssssuGAGAuuuuGG.sssTTA
  consensus/70%                   ......................GAuGCTGTATTssssuGAGAuuuuGG.sssTTA

                   cov    pid 2721        .         .         :         .         .
1 mmAtp7a3pUTR   100.0% 100.0%    CAGTTGGCCACTGGTTTAA-GTAATGGGCCACTGCTCAGAACAGGTTTTGTAAGT
2 hsATP7A3pUTR    84.7%  47.6%    CAGTTAGTAGCTAGCTTTAAGTCAGGAGTTAGT---A--------ATGAGAAATT
  consensus/100%                  CAGTTuGssuCTuGsTTsA.GTsAsGuGssAsT...s........sTssGsAAsT
  consensus/90%                   CAGTTuGssuCTuGsTTsA.GTsAsGuGssAsT...s........sTssGsAAsT
  consensus/80%                   CAGTTuGssuCTuGsTTsA.GTsAsGuGssAsT...s........sTssGsAAsT
  consensus/70%                   CAGTTuGssuCTuGsTTsA.GTsAsGuGssAsT...s........sTssGsAAsT

                   cov    pid 2801        .         .         .         .         :
1 mmAtp7a3pUTR   100.0% 100.0%    TATATACACATGTATATACACACACACACATACATGCATACAGAGAGAGACAGCC
2 hsATP7A3pUTR    84.7%  47.6%    CACATACATATAT--ATATACACACATATATATGTACATACACATACATACATAT
  consensus/100%                  sAsATACAsATuT..ATAsACACACAsAsATAsuTuCATACAsAsAsAsACAsss
  consensus/90%                   sAsATACAsATuT..ATAsACACACAsAsATAsuTuCATACAsAsAsAsACAsss
  consensus/80%                   sAsATACAsATuT..ATAsACACACAsAsATAsuTuCATACAsAsAsAsACAsss
  consensus/70%                   sAsATACAsATuT..ATAsACACACAsAsATAsuTuCATACAsAsAsAsACAsss

                   cov    pid 2881        .         9         .         .         .
```

```
1 mmAtp7a3pUTR    100.0% 100.0%        ---AGAGAGGGGGAGAGAGCATACATCTTTCGAGGTTCTTTATACTTTCCTTGC
2 hsATP7A3pUTR     84.7%  47.6%        ATATTTGAATTTGATATTTGAATATTTGAAGTTCTCTATTTATATTTCATTTAT
  consensus/100%                       ...sssGAusssGAsAssssAssssTssssssssssTsTTTATAsTTsssTTus
  consensus/90%                        ...sssGAusssGAsAssssAssssTssssssssssTsTTTATAsTTsssTTus
  consensus/80%                        ...sssGAusssGAsAssssAssssTssssssssssTsTTTATAsTTsssTTus
  consensus/70%                        ...sssGAusssGAsAssssAssssTssssssssssTsTTTATAsTTsssTTus

                     cov    pid 2961            .         .         .         0         .
1 mmAtp7a3pUTR    100.0% 100.0%        GTTGTCCCTGTCAGAAGGCGCCATGGACACTTTGCTGTCCAGTGCTAATGCGGA
2 hsATP7A3pUTR     84.7%  47.6%        ATGCCCTTAGTCTGAAGATAACAAGGATACTTTAATATCCAGTGCCGGTTCAGA
  consensus/100%                       uTsssCsssGTCsGAAGususCAsGGAsACTTTusTuTCCAGTGCsuuTsCuGA
  consensus/90%                        uTsssCsssGTCsGAAGususCAsGGAsACTTTusTuTCCAGTGCsuuTsCuGA
  consensus/80%                        uTsssCsssGTCsGAAGususCAsGGAsACTTTusTuTCCAGTGCsuuTsCuGA
  consensus/70%                        uTsssCsssGTCsGAAGususCAsGGAsACTTTusTuTCCAGTGCsuuTsCuGA

                     cov    pid 3041            :         .         .         .         .
1 mmAtp7a3pUTR    100.0% 100.0%        AAATATCCAAAGATGCCTTTTGAATTTAAGATTAA--AACACAGCTAGAATATT
2 hsATP7A3pUTR     84.7%  47.6%        AAATATCCAAAGATGCCTTTTGAATTTCAAAGATTAAAACACAGCTAGAGTAGT
  consensus/100%                       AAATATCCAAAGATGCCTTTTGAATTTsAuAssss..AACACAGCTAGAuTAsT
  consensus/90%                        AAATATCCAAAGATGCCTTTTGAATTTsAuAssss..AACACAGCTAGAuTAsT
  consensus/80%                        AAATATCCAAAGATGCCTTTTGAATTTsAuAssss..AACACAGCTAGAuTAsT
  consensus/70%                        AAATATCCAAAGATGCCTTTTGAATTTsAuAssss..AACACAGCTAGAuTAsT

                     cov    pid 3121            .         .         :         .         .
1 mmAtp7a3pUTR    100.0% 100.0%        AGTTGTTAGGTGTGAAATAGAAATGTTGCTTTTCCTACTTTAGTATCACCATTT
2 hsATP7A3pUTR     84.7%  47.6%        TGTGTCATTTGTGAAAATAGAAACGTTATTTTTCCTAGTTTAGTATCAACATTT
  consensus/100%                       sGTssssssssssuAAATAGAAAsGTTusTTTTCCTAsTTTAGTATCAsCATTT
  consensus/90%                        sGTssssssssssuAAATAGAAAsGTTusTTTTCCTAsTTTAGTATCAsCATTT
  consensus/80%                        sGTssssssssssuAAATAGAAAsGTTusTTTTCCTAsTTTAGTATCAsCATTT
  consensus/70%                        sGTssssssssssuAAATAGAAAsGTTusTTTTCCTAsTTTAGTATCAsCATTT

                     cov    pid 3201            .         .         .         .         :
1 mmAtp7a3pUTR    100.0% 100.0%        AACAAATAATTTTATATTATGCTTTAATTTTTTAAAAATTTTCTTCATTCAGAT
2 hsATP7A3pUTR     84.7%  47.6%        AACAAGTAATTTTATATTGTGCTTTAATTTTTTAAAAAGTATTCT---TTATTC
  consensus/100%                       AACAAuTAATTTTATATTuTGCTTTAATTTTTTAAAAAsTsTssT...TsAsss
  consensus/90%                        AACAAuTAATTTTATATTuTGCTTTAATTTTTTAAAAAsTsTssT...TsAsss
  consensus/80%                        AACAAuTAATTTTATATTuTGCTTTAATTTTTTAAAAAsTsTssT...TsAsss
  consensus/70%                        AACAAuTAATTTTATATTuTGCTTTAATTTTTTAAAAAsTsTssT...TsAsss

                     cov    pid 3281            .         3         .         .         .
1 mmAtp7a3pUTR    100.0% 100.0%        CTATAGACACGATGTTTCCAAAAATGTGACTGTGCATATATTTTCTGTACACAG
2 hsATP7A3pUTR     84.7%  47.6%        CTGTAGACAAGATGTTTCCAAAACTTTAAGTGCGCATATCTTTTCTGTATACAG
  consensus/100%                       CTuTAGACAsGATGTTTCCAAAAsTsTuAsTGsGCATATsTTTTCTGTAsACAG
  consensus/90%                        CTuTAGACAsGATGTTTCCAAAAsTsTuAsTGsGCATATsTTTTCTGTAsACAG
  consensus/80%                        CTuTAGACAsGATGTTTCCAAAAsTsTuAsTGsGCATATsTTTTCTGTAsACAG
  consensus/70%                        CTuTAGACAsGATGTTTCCAAAAsTsTuAsTGsGCATATsTTTTCTGTAsACAG

                     cov    pid 3361            .         .         .         4         .
1 mmAtp7a3pUTR    100.0% 100.0%        GATTTTTCCAT--TACGTGTAGATTCTGTTTTAAAATTATTTTTCATATTTATT
2 hsATP7A3pUTR     84.7%  47.6%        GAATTTTTTCTATTACTTATGGATCATGCTTTAAAATGATTTTTCTTGATA---
  consensus/100%                       GAsTTTTsssT..TACsTuTuGATssTGsTTTAAAATsATTTTTCsTusTs...
  consensus/90%                        GAsTTTTsssT..TACsTuTuGATssTGsTTTAAAATsATTTTTCsTusTs...
  consensus/80%                        GAsTTTTsssT..TACsTuTuGATssTGsTTTAAAATsATTTTTCsTusTs...
  consensus/70%                        GAsTTTTsssT..TACsTuTuGATssTGsTTTAAAATsATTTTTCsTusTs...

                     cov    pid 3441            :         .         .         .         .
1 mmAtp7a3pUTR    100.0% 100.0%        TTTTTTTGTATGAGGCATCTGGTTAAAGCTTAAGCTGCTTCTCTTCTTCCTCTT
2 hsATP7A3pUTR     84.7%  47.6%        TTTCTCTGGGTGAGGCATCTGGTTACTGGTTATTTTAAAAA-------------
  consensus/100%                       TTTsTsTGsuTGAGGCATCTGGTTAssGsTTAsssTusssss.............
```

```
   consensus/90%                      TTTsTsTGsuTGAGGCATCTGGTTAssGsTTAsssTussss..............
   consensus/80%                      TTTsTsTGsuTGAGGCATCTGGTTAssGsTTAsssTussss..............
   consensus/70%                      TTTsTsTGsuTGAGGCATCTGGTTAssGsTTAsssTussss..............

                    cov    pid 3521            .         .         :         .         .
 1 mmAtp7a3pUTR   100.0% 100.0%       CCCTCTTCCTTCTCTTCTTCCTTCTCCTTTTTATACAGACATTCCTCATAATCAC
 2 hsATP7A3pUTR    84.7%  47.6%       ------------------------------GAATAAAGACATTCCTGGAATCACT
   consensus/100%                     ..............................ssATAsAGACATTCCTsusAsssss
   consensus/90%                      ..............................ssATAsAGACATTCCTsusAsssss
   consensus/80%                      ..............................ssATAsAGACATTCCTsusAsssss
   consensus/70%                      ..............................ssATAsAGACATTCCTsusAsssss

                    cov    pid 3601            .         .         .         .         :
 1 mmAtp7a3pUTR   100.0% 100.0%       ATTAAAAAACATACTTCTTTACCAAATATCGATAGATAGATAGATAGATAGATAG
 2 hsATP7A3pUTR    84.7%  47.6%       -------------TTTCTTTTCTAC------------------------------
   consensus/100%                     .............sTTCTTTsCsAs..............................
   consensus/90%                      .............sTTCTTTsCsAs..............................
   consensus/80%                      .............sTTCTTTsCsAs..............................
   consensus/70%                      .............sTTCTTTsCsAs..............................

                    cov    pid 3681            .         7         .         .         .
 1 mmAtp7a3pUTR   100.0% 100.0%       TAGATAGATAGATAGATAGATAGATTGATTCATGCCAAATCTTTACCAACTTTTA
 2 hsATP7A3pUTR    84.7%  47.6%       TTATTTTACAAAATAGAAAAAATATAGATTCATGCCAAATTATTACCTATTTTTA
   consensus/100%                     TsusTssAsAuAssuusAuAsAsATsGATTCATGCCAAATssTTACCsAsTTTTA
   consensus/90%                      TsusTssAsAuAssuusAuAsAsATsGATTCATGCCAAATssTTACCsAsTTTTA
   consensus/80%                      TsusTssAsAuAssuusAuAsAsATsGATTCATGCCAAATssTTACCsAsTTTTA
   consensus/70%                      TsusTssAsAuAssuusAuAsAsATsGATTCATGCCAAATssTTACCsAsTTTTA

                    cov    pid 3761            .         .         .         8         .
 1 mmAtp7a3pUTR   100.0% 100.0%       CTGGTATGAGTAGCTAGCTATAACACAAAGCATGAATTCTTATTTCTCAATCACA
 2 hsATP7A3pUTR    84.7%  47.6%       CTGGTATGAGTAACCAGATAGAGCACAAAGCATGAGTTCTTGTTCTTCAGTTAAA
   consensus/100%                     CTGGTATGAGTAuCsAGsTAsAuCACAAAGCATGAuTTCTTuTTssTCAuTsAsA
   consensus/90%                      CTGGTATGAGTAuCsAGsTAsAuCACAAAGCATGAuTTCTTuTTssTCAuTsAsA
   consensus/80%                      CTGGTATGAGTAuCsAGsTAsAuCACAAAGCATGAuTTCTTuTTssTCAuTsAsA
   consensus/70%                      CTGGTATGAGTAuCsAGsTAsAuCACAAAGCATGAuTTCTTuTTssTCAuTsAsA

                    cov    pid 3841            :         .         .         .         .
 1 mmAtp7a3pUTR   100.0% 100.0%       TAATTGACTTTTGTAGCCAGAGCCAGGTGTCTCAACCTTGTGTTTTTAAAGAAAT
 2 hsATP7A3pUTR    84.7%  47.6%       CAAATGCCTTTTGTAGCCAAAACCAGGCGTCTCAACCTTACGTTTTTAGTTAAAC
   consensus/100%                     sAAsTGsCTTTTGTAGCCAuAuCCAGGsGTCTCAACCTTusGTTTTTAussAAAs
   consensus/90%                      sAAsTGsCTTTTGTAGCCAuAuCCAGGsGTCTCAACCTTusGTTTTTAussAAAs
   consensus/80%                      sAAsTGsCTTTTGTAGCCAuAuCCAGGsGTCTCAACCTTusGTTTTTAussAAAs
   consensus/70%                      sAAsTGsCTTTTGTAGCCAuAuCCAGGsGTCTCAACCTTusGTTTTTAussAAAs

                    cov    pid 3921            .         .         :         .         .
 1 mmAtp7a3pUTR   100.0% 100.0%       ----------TTAGAATCCAAAACAGGAGCGGGCAACTTTGTGCAATATTTCGTT
 2 hsATP7A3pUTR    84.7%  47.6%       CATTGATTGTTTGGTACCGAAAACAGCAGTGGACGATGTTGTGCAATATCCATCT
   consensus/100%                     ..........TTuGsAsCsAAAACAGsAGsGGuCuAssTTGTGCAATATsssssT
   consensus/90%                      ..........TTuGsAsCsAAAACAGsAGsGGuCuAssTTGTGCAATATsssssT
   consensus/80%                      ..........TTuGsAsCsAAAACAGsAGsGGuCuAssTTGTGCAATATsssssT
   consensus/70%                      ..........TTuGsAsCsAAAACAGsAGsGGuCuAssTTGTGCAATATsssssT

                    cov    pid 4001            .         .         .         .         :
 1 mmAtp7a3pUTR   100.0% 100.0%       TGTTTTTTCTCATGTGCATGTAATTGATATTTCTGCCGAAGATGTGCCTTCAACT
 2 hsATP7A3pUTR    84.7%  47.6%       TGTTTTTCATAAGCATGTAATTGATCATATTTCTGCCAAGGATGTGCCTTCAACT
   consensus/100%                     TGTTTTTssTsAssssssssuTsusTsATATTTCTGCCuAuGATGTGCCTTCAACT
   consensus/90%                      TGTTTTTssTsAssssssssuTsusTsATATTTCTGCCuAuGATGTGCCTTCAACT
   consensus/80%                      TGTTTTTssTsAssssssssuTsusTsATATTTCTGCCuAuGATGTGCCTTCAACT
   consensus/70%                      TGTTTTTssTsAssssssssuTsusTsATATTTCTGCCuAuGATGTGCCTTCAACT
```

```
                    cov    pid 4081          .         1         .         .         .
1 mmAtp7a3pUTR   100.0% 100.0%      AAAAATGTTGTCAAGTGTCGCTGTGTTAAGTCATCCAGCCCCCTCTATCAGCAGA
2 hsATP7A3pUTR    84.7%  47.6%      -------------------------------------------------------
  consensus/100%                    .......................................................
  consensus/90%                     .......................................................
  consensus/80%                     .......................................................
  consensus/70%                     .......................................................

                    cov    pid 4161          .         .         .         2         .
1 mmAtp7a3pUTR   100.0% 100.0%      GTGGTTGTCTGGCTGTTGTTTCAGCTGTAAATCCGTGCTGTGTCAGGATCAGAGC
2 hsATP7A3pUTR    84.7%  47.6%      -------------------------------------------------------
  consensus/100%                    .......................................................
  consensus/90%                     .......................................................
  consensus/80%                     .......................................................
  consensus/70%                     .......................................................

                    cov    pid 4241          :         .         .         .         .
1 mmAtp7a3pUTR   100.0% 100.0%      CAAAAATCTGTTGTCTCTCAGATCCCGCTGCCCTGCTGCTGTCACTTAGAACACC
2 hsATP7A3pUTR    84.7%  47.6%      -------------------------------------------------------
  consensus/100%                    .......................................................
  consensus/90%                     .......................................................
  consensus/80%                     .......................................................
  consensus/70%                     .......................................................

                    cov    pid 4321          .         .         :         .      ] 4367
1 mmAtp7a3pUTR   100.0% 100.0%      TCTCCTTTGTAGTGTCTAATAAATTCTTGAGACGGTGTCTCCCCTCC
2 hsATP7A3pUTR    84.7%  47.6%      -----------------------------------------------
  consensus/100%                    ...............................................
  consensus/90%                     ...............................................
  consensus/80%                     ...............................................
  consensus/70%                     ...............................................
```

MView 1.63, Copyright © 1997-2018 Nigel P. Brown

Supplementary Figure 3: Substantial conservation is observed in murine and human *ATP7A* 3' UTRs. Multiple sequence alignment and consensus sequence of murine *Atp7a* 3' UTR (NM_001109757) and human *ATP7A* 3' UTR (NM_001282224) as visualized in MView.

Figure S4

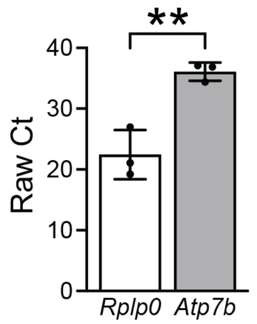

Supplementary Figure 4: *Atp7b* is not detected in myoblasts. Raw Ct values for qRT-PCR using primers to *Rplp0* and *Atp7b* in myoblasts showing Ct values for *Atp7b* at nearly undetectable levels above 30. Shown is mean ± standard deviation for n = 3-4 experiments. Statistical significance was determined by t-test (** $p < 0.01$).

Figure S5

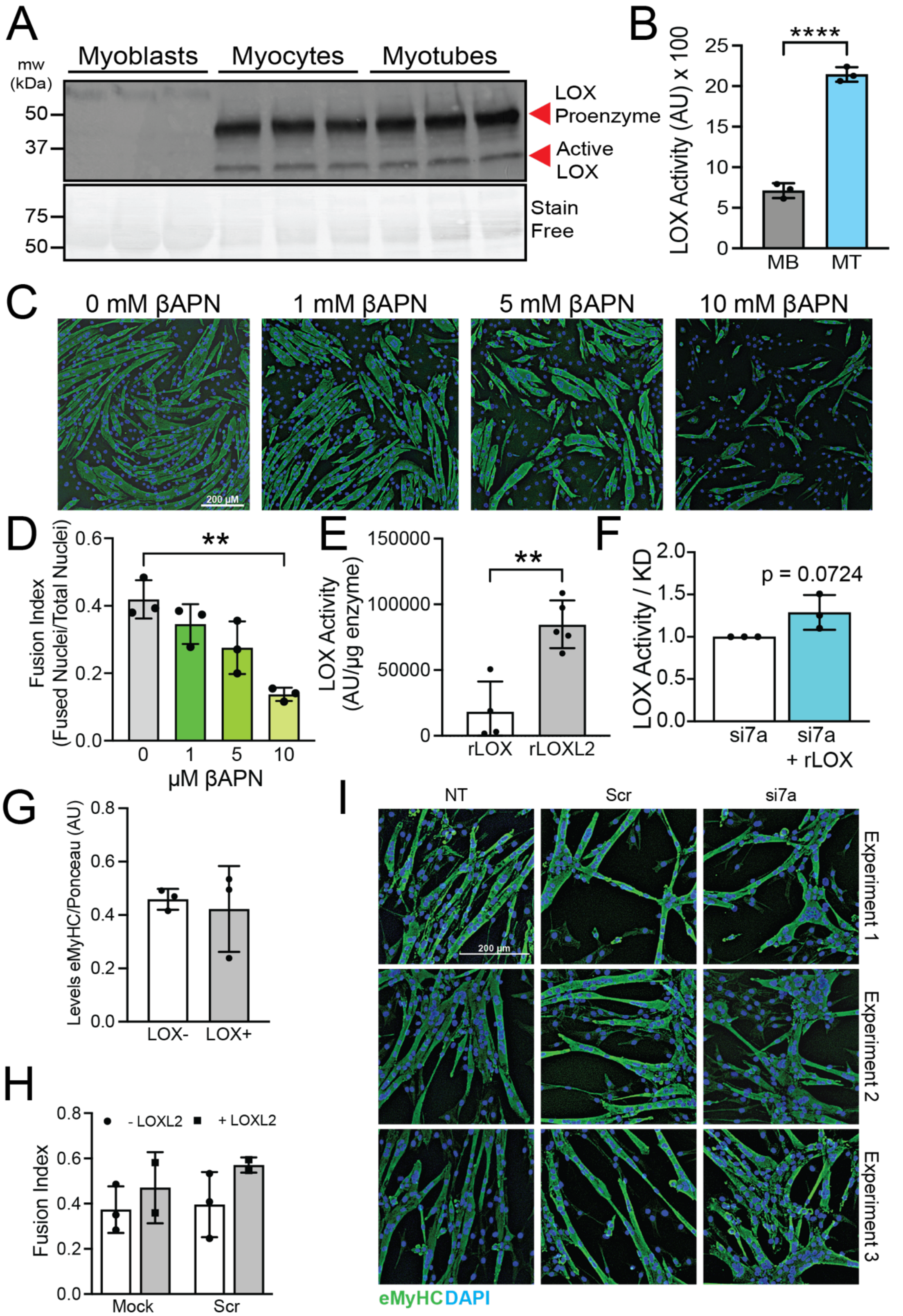

A
mw (kDa)
Myoblasts
Myocytes
Myotubes
50
37
75
50
LOX Proenzyme
Active LOX
Stain Free
B
LOX Activity (AU) x 100
****
MB
MT
C
0 mM βAPN
1 mM βAPN
5 mM βAPN
10 mM βAPN
200 μM
D
Fusion Index (Fused Nuclei/Total Nuclei)
**
μM βAPN
E
LOX Activity (AU/μg enzyme)
**
rLOX
rLOXL2
F
LOX Activity / KD
p = 0.0724
si7a
si7a + rLOX
G
Levels eMyHC/Ponceau (AU)
LOX-
LOX+
H
Fusion Index
- LOXL2
+ LOXL2
Mock
Scr
I
NT
Scr
si7a
200 μm
Experiment 1
Experiment 2
Experiment 3
eMyHC
DAPI

Supplementary Figure 5: LOX activity is required for C2C12 myoblast differentiation. ***A)*** Immunoblot of conditioned medium from C2C12 myoblasts (MB), myocytes (MC), and myotubes (MT) lysates probed with an antibody to LOX and with total protein shown using Stain-free gel technology. The LOX proenzyme is detected at ~45 kDa while process and active LOX is detected at ~30 kDa. ***B)*** LOX activity shown as arbitrary units (AU) x 100 showing increased activity in MT relative to MB. ***C)*** C2C12 myoblasts were induced to differentiate in the presence of 0, 1, 5, or 10 mM of the LOX inhibitor βAPN and stained with an antibody to eMyHC and DAPI to visualize nuclei. ***D)*** Fusion index quantification revealing a significant decrease in myotube formation caused by LOX inhibition with βAPN. ***E)*** In vitro activity of recombinant human LOX (rLOX) and recombinant human LOXL2 (rLOXL2) as AU x 100. ***F)*** Total LOX activity in *Atp7a* knockdown myotubes (si7a) versus knockdown myotubes with added rLOX (si7a + LOX) reported as fold change of knockdown. ***G)*** Immunoblot quantification showing no change in levels of eMyHC in Scr myotubes with or without added LOX. ***H)*** Fusion index showing no change in non-treated or Scr myotubes with or without added LOXL2. ***I)*** Additional representative images of NT, Scr, and si7a myoblasts differentiated on Matrigel showing three independent experiments stained with an antibody to eMyHC with nuclei stained with DAPI. Bar = 200 µm. Shown is the mean ± standard deviation for n = 3 experiments. Statistical significance was determined using t-test or one-way ANOVA (** $p < 0.01$, **** $p < 0.0001$).

Supplementary Table 1: Half-life of *Atp7a* RNA under various conditions

Condition	T(1/2)	p*
MB	5.37	-
MC	7.74	0.0075
MT	8.86	0.16
MB Cu	18.7	0.0005
MB TEPA	6.56	0.66
* Comparison to untreated MB		

Supplementary Table 2: Predicted protein binding sites in the proximal *ATP7A* 3' UTR

RBP	Prediction	RBS location	Score	PhastCons score	Transcript ID	Context
HuR	Deep bind	chrX:77302350-77302400;+	0.364	0.593	ENST00000343533	UTR3
HuR	Deep bind	chrX:77302650-77302700;+	0.21	0.419	ENST00000343533	UTR3
HNRNPA1	TESS	chrX:77302300-77302350;0	0.198	0.703	ENST00000343533	UTR3
HNRNPH1	TESS	chrX:77302550-77302600;0	0.751	1.638	ENST00000343533	UTR3
HNRNPH1	FIMO	chrX:77304800-77304850;0	0.421	0.563	ENST00000341514	UTR3
HNRNPH1	FIMO	chrX:77302400-77302450;0	0.26	0.788	ENST00000350425	UTR3
HNRNPCL1	TESS, FIMO	chrX:77302350-77302400;0	0.364	0.593	ENST00000343533	UTR3
HNRNPCL1	FIMO	chrX:77302250-77302300;0	0.425	0.801	ENST00000343533	UTR3
RBFOX1	Deep bind	chrX:77302400-77302450;+	0.26	0.788	ENST00000343533	UTR3
PABPC5	TESS, FIMO	chrX:77302400-77302450;0	0.26	0.788	ENST00000343533	UTR3
PTBP1	TESS, FIMO	chrX:77302950-77303000;0	0.119	0.482	ENST00000343533	UTR3
HNRNPC	TESS, FIMO	chrX:77302350-77302400;0	0.364	0.593	ENST00000343533	UTR3
HNRNPC	FIMO	chrX:77302250-77302300;0	0.425	0.801	ENST00000343533	UTR3
HNRNPC	FIMO	chrX:77302150-77302200;0	0.113	0.488	ENST00000343533	UTR3
PABPC4	TESS, FIMO	chrX:77302350-77302400;0	0.364	0.593	ENST00000343533	UTR3
QKI	TESS, FIMO	chrX:77302250-77302300;0	0.425	0.801	ENST00000343533	UTR3
YBX2	TESS	chrX:77302250-77302300;0	0.425	0.801	ENST00000343533	UTR3
HNRNPA1L2	TESS, FIMO	chrX:77302150-77302200;0	0.113	0.488	ENST00000343533	UTR3
ESRP2	FIMO	chrX:77302500-77302550;0	0.056	0.456	ENST00000343533	UTR3

Supplementary Table 3: Primary antibodies used in this study

Antibody	Manufacturer information	Experiment	Dilution
eMyHC	DSHB F1.652 hybridoma supernatant	Stain	1:10
eMyHC	DSHB F1.652 bioreator supernatant	Blot	1:10,000
MYOG	DSHB FD5 hybridoma supernatant	Blot	1:100
CCS	Santa Cruz (H-7) sc-55561	Blot	1:1000
ATP7A	Wang, et. al *Plos One* , 2012	Blot	1:3000
ATOX1	Proteintech 226411-1-AP	Blot	1:1000
LOX	Novus Biologicals #NB1002527SS	Blot	1:1000

Supplementary Table 4: Primers used in this study

Primer name	Sequence
Atp7a F	*CCAAGGGTGTGACTGGTGTT*
Atp7a R	*GGCACTCACCACAGATGGAA*
Myc F	*CGCGATCAGCTCTCCTGAAA*
Myc R	*AAGTTCACGTTGAGGGGCAT*
Atp7b F	*CCCAGGAAGAACTTGGCGTC*
Atp7b R	*TTTGGCTGTGACCTGTCTCTC*
Myh3 F	*TCTCTGTCACAGTCAGAGGTGT*
Myh3 R	*TTTTCCGACTTGCGGAGGAA*
Myog F	*CACTGGAGTTCGGTCCCAA*
Myog R	*TGTGGGCGTCTGTAGGGTC*
Acta1 F	*GCGACCTCACTGACTACCTG*
Acta1 R	*GATGTCGCGCACAATCTCAC*
